# Artificial intelligence supporting cancer patients across Europe—The ASCAPE project

**DOI:** 10.1371/journal.pone.0265127

**Published:** 2022-04-21

**Authors:** Lazaros Tzelves, Ioannis Manolitsis, Ioannis Varkarakis, Mirjana Ivanovic, Miltiadis Kokkonidis, Cristina Sabater Useros, Thanos Kosmidis, Montserrat Muñoz, Imma Grau, Manos Athanatos, Anamaria Vizitiu, Konstantinos Lampropoulos, Tzortzia Koutsouri, Dimitra Stefanatou, Konstantinos Perrakis, Christina Stratigaki, Serge Autexier, Paris Kosmidis, Antonis Valachis

**Affiliations:** 1 National and Kapodistrian University of Athens Faculty of Medicine Marousi, 2nd Department of Urology, Athens, Greece; 2 Faculty of Sciences, University of Novi Sad, Novi Sad, Serbia; 3 Intrasoft International Ltd., Luxembourg, Luxembourg; 4 ATOS SE, Madrid, Spain; 5 CareAcross Ltd, London, United Kingdom; 6 Department of Medical Oncology, Hospital Clinic of Barcelona, Barcelona, Spain; 7 Foundation for Research and Technology-Hellas (FORTH), Heraklion, Crete, Greece; 8 Siemens SRL, Bucuresti, Romania; 9 Department of Electrical and Computer Engineering, University of Patras, Patras, Greece; 10 Sphynx Technology Solutions AG, Zug, Switzerland; 11 Arthur’s Legal, Amsterdam, The Netherlands; 12 UBITECH Research Department, UBITECH Ltd., Athens, Greece; 13 German Research Center for Artificial Intelligence (DFKI), Bremen, Germany; 14 Second Medical Oncology Department, Hygeia Hospital, Athens, Greece; 15 Department of Oncology, Faculty of Medicine and Health, Örebro University, Örebro, Sweden; PLOS: Public Library of Science, UNITED KINGDOM

## Abstract

**Introduction:**

Breast and prostate cancer survivors can experience impaired quality of life (QoL) in several QoL domains. The current strategy to support cancer survivors with impaired QoL is suboptimal, leading to unmet patient needs. ASCAPE aims to provide personalized- and artificial intelligence (AI)-based predictions for QoL issues in breast- and prostate cancer patients as well as to suggest potential interventions to their physicians to offer a more modern and holistic approach on cancer rehabilitation.

**Methods and analyses:**

An AI-based platform aiming to predict QoL issues and suggest appropriate interventions to clinicians will be built based on patient data gathered through medical records, questionnaires, apps, and wearables. This platform will be prospectively evaluated through a longitudinal study where breast and prostate cancer survivors from four different study sites across the Europe will be enrolled. The evaluation of the AI-based follow-up strategy through the ASCAPE platform will be based on patients’ experience, engagement, and potential improvement in QoL during the study as well as on clinicians’ view on how ASCAPE platform impacts their clinical practice and doctor-patient relationship, and their experience in using the platform.

**Ethics and dissemination:**

ASCAPE is the first research project that will prospectively investigate an AI-based approach for an individualized follow-up strategy for patients with breast- or prostate cancer focusing on patients’ QoL issues. ASCAPE represents a paradigm shift both in terms of a more individualized approach for follow-up based on QoL issues, which is an unmet need for cancer survivors, and in terms of how to use Big Data in cancer care through democratizing the knowledge and the access to AI and Big Data related innovations.

**Trial registration:**

Trial Registration on clinicaltrials.gov: NCT04879563.

## Background

The number of cancer survivors is steadily increased over time due to the advances in cancer diagnosis and treatment strategies (1). Considering the high incidence of breast- and prostate cancer in women and men, respectively and the increased survival for these malignancies over time, it is not surprising that these are the most prevalent types of neoplasms in both genders [1].

Many cancer patients experience adverse effects of cancer or associated treatment, which can considerably decrease health-related quality of life (QoL) [2,3]. The current strategy of supporting cancer patients/survivors does not meet their needs due to the limited personalized-based approach in rehabilitation plan and the lack of healthcare, financial and other resources [4–7].

The recent advances in Artificial Intelligence (AI) and Big Data analytics, give the opportunity to improve many aspects of healthcare [8]. In oncology, AI has an emerging role in several clinical aspects including screening and diagnosis regarding both radiology and pathology, prognostic prediction and risk stratification, as well as treatment selection and response prediction [9]. However, the role of AI in follow-up of cancer survivors with focus on long-term toxicity and health-related QoL has not been studied in a prospective manner adequately.

ASCAPE (Artificial intelligence Supporting CAncer Patients across Europe) is a collaborative research project involving 15 partners from 7 countries, including academic medical centers, SMEs (small and medium-sized enterprises), research centers and universities, aiming to leverage the recent advances in Big Data and AI (Artificial Intelligence) to support cancer patients’ QoL and health status. Specifically, ASCAPE aims to provide personalized- and AI-based predictions for QoL issues in breast- and prostate cancer patients as well as suggest potential interventions to their physicians.

## Methods/Design

### Study design and setting

Four study sites comprise the ASCAPE project, with each one of them planned to run the protocol with several modifications according to their setting and current practice.

Barcelona study site (Hospital Clinic de Barcelona, iSYS Foundation) will include breast cancer survivors currently being followed up after curative treatment. Patients will be randomly assigned in two groups (control vs. intervention), for a total follow-up period of 12 months. Both groups will benefit from the ASCAPE platform for AI-based predictions, but the intervention group will also use a locally designed smartphone application (Xemio), which will be studied upon its effect on patients’ QoL. The Barcelona study site will also involve Primary Care Centres in Barcelona, considering that primary care setting is in part responsible for follow-up of breast cancer survivors.

Örebro and Uppsala University Hospitals (ORB/Uppsala) will include, in their prospective single-arm longitudinal study cohort, breast- (ORB/Uppsala) and prostate (ORB) cancer patients after curative treatment and will incorporate the ASCAPE platform into the current follow-up practice during a 12-month follow-up period.

Athens study site (National and Kapodistrian University of Athens Sismanogleio General Hospital, Urology Department; NKUA) will recruit patients diagnosed with localized prostate cancer and planned for treatment with radical prostatectomy, in a prospective single-arm longitudinal study. The patients will be followed using the ASCAPE platform for 12 months.

CareAcross, an online platform that focuses on supporting cancer patients through education about their condition, will prospectively enroll breast- and prostate cancer patients, to provide them with a version of its current personalized services including the AI-based prediction models from ASCAPE.

## Subject selection and recruitment

The patient selection process varies among the different study sites. Three Hospitals (NKUA, ORB, Uppsala) will include patients with newly diagnosed breast- (ORB, Uppsala) or prostate cancer (NKUA, ORB) who are eligible for curative treatment with surgery (breast cancer, prostate cancer in NKUA) or radiotherapy (prostate cancer; ORB). One Hospital (Barcelona) will include breast cancer survivors (at least 12 months after surgery or chemotherapy) with follow-up through the Hospital. Finally, CareAcross will include patients with breast- or prostate cancer through its online platform for patients seeking for the CareAcross services.

Considering the differences among the study sites, the patients’ inclusion and exclusion criteria also differ to some extent.

For breast cancer patients, ORB and Uppsala includes breast cancer patients without clinical evidence of metastatic disease, able for curative treatment with surgery with or without oncological treatment. Barcelona includes patients with prior early breast cancer who are at follow-up with at least 12 months after surgery or chemotherapy (whichever occurred last). CareAcross includes all patients with breast cancer diagnosis (as per self-reported) irrespective of stage and treatment.

For prostate cancer patients, NKUA and ORB includes prostate cancer patients without clinical evidence of metastatic disease, able for curative treatment with surgery with or without oncological treatment (NKUA) or radiotherapy (with or without prior surgery) irrespectively the type of radiotherapy (external radiotherapy, brachytherapy, or combination). CareAcross includes all patients with prostate cancer diagnosis (as per self-reported) irrespective of stage and treatment.

Signed informed consent before study inclusion is mandatory for all included patients from all participating sites.

Some common exclusion criteria for both breast- and prostate cancer and among all study sites do exist including inability to give informed consent, inability / no access to smartphones, applications or internet services, and patients with known medical history of allergy to the wearable material.

### Study procedures

The procedure followed by each study site differs according to the local follow-up algorithms and practices, but all four study sites will eventually use the ASCAPE platform for AI-based predictions of QoL issues as a part of their follow-up strategy for eligible patients.

Barcelona study site will combine ASCAPE platform with the Xemio application (https://www.xemio.org/es/). Eligible patients will be randomized to either intervention arm (patients with access to the Xemio app) or control arm (patients without access to the Xemio app). The study will last for 12 months, and every 3 months, all participants will receive the same QoL questionnaires via e-mail (control arm) or application notifications (intervention arm). All data from both arms will be transferred to a platform connected to ASCAPE and will simultaneously contribute to ASCAPE´s development and evaluation as a follow-up tool. Treating physicians will advise the ASCAPE-based predictions for each patient and decide after discussing with the patient if the proposed intervention should be implemented for the clinical scenario presented.

In ORB/Uppsala study site, the ASCAPE platform will be totally integrated into the current follow-up strategy. Eligible patients will be followed using QoL questionnaires (every 3 months for 12 months) and wearables to gather active monitoring data for 12 months. These data along with baseline characteristics and medically relevant information for eligible patients, will be used by the ASCAPE-platform for individualized AI-based predictions that will be communicated to the patients by treating physicians. Any proposed intervention based on ASCAPE predictions will be decided by the treating physician and the patient based on the current standard of care and will be evaluated regarding its effectiveness during the follow-up.

In NKUA study site, the ASCAPE platform will be gradually integrated into the current follow-up strategy. After agreement to participate, all patients will be provided with a wearable device for a 14-day period of use prior to surgical treatment, to record baseline active monitoring data preoperatively. At the time of admission to the Hospital, baseline data for each eligible patient will be collected. The wearable device will be worn by the patients for a total of 12 months. The follow up will be performed at 3-monthly intervals with the use of QoL questionnaires. All patient data will be collected on paper at the time of admission and ASCAPE platform will sync with a specifically designed website which will be used to collect relevant patient data electronically. After accessing the website, patients will fill out the follow up questionnaires, online, using a unique personal code. The ASCAPE platform will offer predictions and suggestions for interventions. As in ORB/Uppsala study site, any proposed intervention based on ASCAPE predictions will be decided by the treating physician and the patient based on the current standard of care and will be evaluated regarding its effectiveness during the follow-up.

In CareAcross study site, patients will, after the electronic agreement and consent, use the CareAcross platform to report their QoL issues, while some will also use a wearable device. Moreover, there will be a corresponding clinician to monitor the whole process and identify any relevant interventions suggested by ASCAPE and consequently, communicate them with the patients individually. Furthermore, this clinician will have access to the ASCAPE platform through which individualized AI-based predictions for QoL issues will be available during a three-month period.

**Fig 1:**
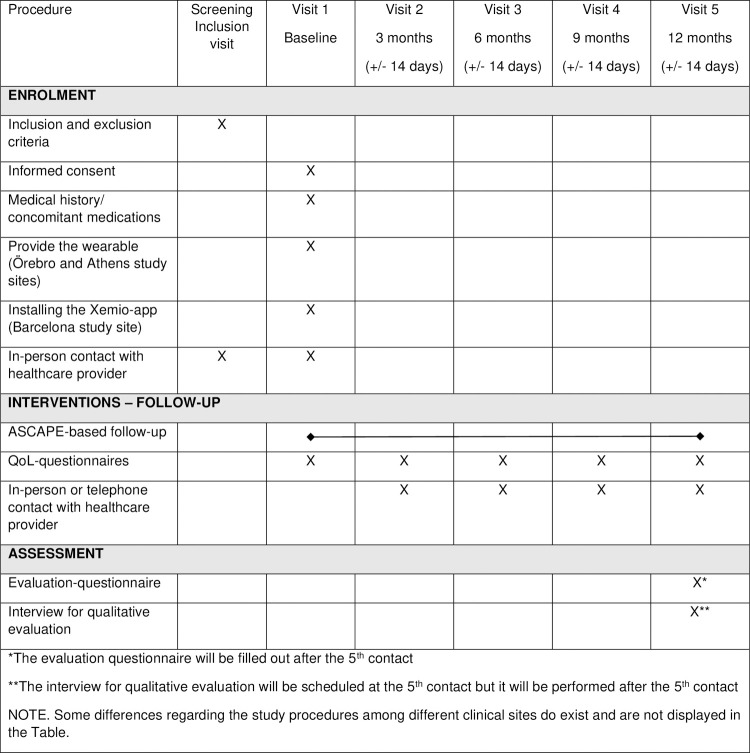
Generic flowchart of study procedures corresponding to ASCAPE-based follow-up strategy.

A generic flowchart of study procedures for the three clinical study sites corresponding to the ASCAPE follow-up strategy is presented in Fig 1.

### Quality of life issues and measures

The identification of QoL issues to be predicted within ASCAPE for breast and prostate cancer was a multistep procedure that was based on current evidence and clinical expertise. Specifically, a literature search, using the electronic database PubMed, was first performed with the following keywords: QoL issues, QoL aspects, health issues, health problems, side effects, and breast cancer or prostate cancer. No year or language restriction was applied but the searching strategy was limited only to systematic reviews or meta-analyses to capture the cumulative evidence on the topic.

The searching strategy yielded nearly 1,300 potentially eligible articles for breast cancer and 650 for prostate cancer. The first study selection was conducted on the title and abstract and the second on the full text. The eligible studies after the two-step selection process were used to build a list of potential QoL issues for breast and prostate cancer, respectively.

To ensure that the process captured all the potential QoL issues, we compared the lists from the searching strategy with the lists from CareAcross retrospective datasets based on patients’ answers on a web-based platform about their actual QoL issues.

After this process, 51 QoL issues for breast cancer and 47 for prostate cancer were identified and included in the lists. The lists of QoL issues on breast and prostate cancer, respectively were sent to the clinical experts within each clinical partner with instructions to prioritize the QoL issues considering the following aspects: how frequent each QoL issue is, the magnitude of impact on patients’ daily life, and the potential positive impact if the QoL issue can be predicted before it occurs.

This multistep process has led to the identification of 15 QoL issues for breast cancer and 12 for prostate cancer that will be predicted through AI-based models within the context of ASCAPE.

The 15 QoL issues for breast cancer are: anxiety, body changes, body image, cognitive impairment, depression, dry vagina, emotional symptoms (loneliness), fatigue, hot flushes, insomnia, joint pain, local symptoms after surgery, lymphedema, neurotoxicity, and sexual dysfunction.

The 12 QoL issues for prostate cancer are: anxiety, bowel dysfunction, cognitive impairment, depression, erectile dysfunction, fatigue, hot flushes, incontinence, low urinary tract symptoms, loss of libido, musculoskeletal pain, and weight changes.

The following questionnaires will be used to capture different aspects on breast cancer patients’ QoL:

- BREAST-Q (mastectomy or breast conserving therapy) [10] at baseline followed by BREAST-Q postoperative (available only in ORB and Uppsala study sites)
- EORTC QLQ C30 [11]: at baseline and every three months
- EORTC QLQ-BR23 [12]: at baseline and every three months
- Hospital Anxiety and Depression Scale (HADS) [13]: at baseline and every three months
- Disability of Arm, Shoulder and Hand questionnaire (DASH) [14]: every three months (available only in ORB and Uppsala study sites)

The following questionnaires will be used to capture different aspects on prostate cancer patients’ QoL:

- EORTC QLQ C30 [11]: at baseline and every 3 months
- EORTC QLQ-PR25 [15]: at baseline and every 3 months
- Hospital Anxiety and Depression Scale (HADS) [13]: at baseline and every three months
- International Index of Erectile Function (IIEF-5) [16]: at baseline and every three months

Considering the nature of CareAcross study site, the different QoL issues for breast- and prostate cancer will be captured through a single Boolean or Likert-scale input whereas the anxiety and depression aspects will be captured using the GAD7 [17] and PHQ9 [18] questionnaires, respectively.

All questionnaires are extensively validated and are recommended by the Breast Cancer Evaluation Database to Guide Effectiveness (EDGE) Task Force and the Prostate Cancer EDGE Task Force, respectively for the clinical measures of health related QoL [19–22].

### Indicators for QoL issues

Considering the lack of evidence on AI-based approaches in monitoring QoL in cancer patients, the choice of potential indicators for QoL issues within ASCAPE is intentionally wide including all medically relevant information to make sure that all potentially predictors will be available for AI-based modelling.

The medically relevant information to be captured as potential indicators for QoL issues can be categorized as follows: patients’ socioeconomic status, baseline characteristics on patients’ lifestyle factors, baseline characteristics on patients’ health condition, baseline characteristics on patients’ reproductive factors (for breast cancer), family history of cancer, tumour characteristics (clinical and pathological features), treatment strategies (including type of treatment, duration, dates), baseline status on QoL issues.

In addition, active monitoring data (through wearables for ORB/Uppsala, NKUA and CareAcross study sites or mobile app for Barcelona study site) and actual nutritional data (through healthy eating assessment questionnaire and anthropometric measurements such as BMI during follow-up) will also be captured and included as potential indicators for QoL issues.

No laboratory results will be considered as QoL indicators because the current guidelines recommend against routine laboratory testing during the follow-up period in breast and prostate cancer except for PSA for prostate cancer recurrence [23–25].

Apart from medically relevant information and active monitoring data, open data including weather data, environmental data, and financial data from the different areas where included patients are living will be included to the AI-based models.

### Quality of life issues and proposed interventions

All the QoL issues for breast- and prostate cancer that derived from the above-mentioned process, went through further assessment by all clinical partners, to be prioritized as potential QoL issues where ASCAPE platform will propose specific interventions. The aspects considered were the following: frequency of each QoL issue in the population; ability for self-reporting; potential need of clinical examination for proper evaluation; number of potential interventions per QoL issue; timeframe for intervention needed for each QoL issue (urgency or not); risk of using AI for each QoL issue.

That assessment yielded 7 QoL issues for breast cancer and 7 for prostate cancer that were considered suitable for proposing individualized interventions through the ASCAPE platform.

The 7 QoL issues for breast cancer are: anxiety, fatigue, depression, joint pain, neurotoxicity, hot flushes, and weight gain whereas the 7 QoL for prostate cancer are: anxiety, depression, fatigue, incontinence, weight change, sexual dysfunction, and hot flushes.

The interventions that are suitable to tackle the QoL issues from each cancer type and will be proposed by the ASCAPE platform depending on current level of evidence are listed in the S1 and S2 Tables.

The outcome of any intervention(s) that will be implemented to the patients, with intention to resolve specific QoL issues will be evaluated during the follow-up as: resolved; improved; unchanged; deteriorated.

The initial prioritization will be enriched by continuous machine learning on prospective data on the efficacy of interventions applied to the patients for each specific QoL to improve the ability of ASCAPE-platform to propose to healthcare professionals a prioritized list of interventions with higher confidence level.

### IT infrastructure

The ASCAPE will be carried out at different study sites with different pre-existing software and hardware infrastructures.

The Barcelona study site involves not only the Barcelona Hospital Clinic (BHC), but also Primary Care Centers (PCCs) in the region. In addition to other software, such as SAP and a traditional hospital medical records system for BHC, both BHC and the PCCs, all have access to an ontology-based health information system designed and running under the auspices of the BHC [26]. Patients in the Barcelona study site will be able to answer questionnaires via a website. Those using the Xemio app will, additionally, be able to report on any QoL issues as they arise.

The NKUA study will be supported by a new web-based application providing both support to doctors at the involved hospital(s) and an interface for patients to submit their answers to questionnaires.

In addition to the hospital information systems used at the ORB/Uppsala study site, in common with established practice at both sites, the Orebro/Uppsala study site will be supported by a commercially available electronic data capture platform (SmartTrial®) for the purposes of collecting data about and from the patients.

Finally, CareAcross will use its own web platform, providing an ASCAPE version of the questionnaires to its registered users that have been invited based on the relevant inclusion criteria and have opted to participate.

Apart from the Barcelona study site, all other ASCAPE studies will involve the use of wearable devices; specifically certain models of FitBit smart bracelets, which were found to satisfy both the technical and the budgetary requirements set out for the studies.

In all study sites, doctors will have access to the same ASCAPE AI-provided functionality. In the case of the NKUA and Barcelona sites, this functionality will be integrated into the software that doctors normally use at each site, whereas in the ORB/Uppsala and CareAcross sites, the web-based ASCAPE Dashboard for Doctors will be used. On the other hand, the patients will not come in direct contact with ASCAPE and the modalities of data collection from patients are optimized for each site.

The ASCAPE AI functionality is provided by a local ASCAPE Edge Node installed at each site. The local ASCAPE Edge Node can perform AI model training and model inference locally, without the patient data leaving each site’s own IT infrastructure. Model training and model inference are the two primary tasks of Machine Learning (ML)-based AI. Model training can be thought of as akin to creating statistical indicators based on the medically relevant data held for patients. Model inference can be thought of as applying the knowledge captured in the AI models to make a prediction about how a patient’s health status may unfold or about what the best interventions for that particular patient may be. The patient in question may or may not be one of the patients whose data were used to train the models; at any rate, the ability of AI to make predictions about each specific case will depend on the availability of a sufficiently large body of data from patients with similar characteristics and with similar disease trajectories as the patient in question at the time the model inference is to be made. If this requirement is not met, no ML-powered prediction will be offered to the patient’s doctor.

If only the local ASCAPE Edge Nodes were used, ASCAPE AI on each site would have been limited to being able to apply knowledge gained from the site’s patients’ data. The advantage of this approach is that patient data are not shared with third parties. The alternative of different sites sharing data on a traditional Cloud-based AI platform would allow knowledge to be built from the patients of all the participating sites, but at the cost of exposing patient data to a third-party’s infrastructure. This is a compromise that hospitals and health-care providers may be unwilling to make. The ASCAPE Edge-Cloud Platform allows the different sites to collectively build ML models capturing knowledge from medically relevant data of patients from all sites collectively, without directly exposing any site’s patients’ data to the ASCAPE Cloud.

This is made possible by the ASCAPE Edge-Cloud architecture and two recent privacy-focused advances in ML techniques as designed and implemented by the ASCAPE Consortium in the context of that architecture:

- Federated ML, whereby each ASCAPE Edge Node enriches a common, global AI model with knowledge from local data (rather than sending these to the ASCAPE Cloud for the model to be trained there) and the ASCAPE Cloud coordinates the process. Model inference also happens locally without the patient’s data being sent to the ASCAPE Cloud.
- Homomorphically encrypted ML, whereby data are sent encrypted to the ASCAPE Cloud where they are stored and processed without ever being decrypted (as the encryption is based on a secret key not shared with the ASCAPE Cloud). The mathematical properties of the encryption method ensure that the model trained on the encrypted data can be used for model inference (either locally or on the ASCAPE Cloud) on encrypted patient data and the result of the inference will be decryptable with the secret key on the ASCAPE Edge node.

Care is also taken when training data models to minimize the risk of inadvertently leaking information about a specific patient via the process of comparing a version of a model before and after the addition of that patient’s data in the set of data on which the model is trained. As the most successful ML-based AI techniques provide little-to-no easily discernible information about how the came to their predictions, additional techniques that help explain AI results to doctors are also used.

### Data transformation

One of ASCAPE challenges is to combine clinical data from different HIS (Hospital Information System) and provide users with a unified structure and common understanding. To achieve that, a harmonization among pilot sources of information is performed via clinical standards: HL7 FHIR specification and SNOMED CT codification.

HL7 FHIR (Fast Healthcare Interoperability Resources) clinical standard is suitable for being used with real time data medical devices as well with smart phones, and medical apps; and it facilitates communication among systems, and electronic health record-based data sharing, data integration and data management. Data stored following FHIR structure is also codified using SNOMED CT, a medical terminology for expression, comprehension, sharing and interconnection of medical concepts.

The harmonization process is divided into several cyclical steps: (i) design of a common data model identifying and including information of interest from all pilot sites; (ii) structuring the common data model by mapping from original variables of interest into HL7 FHIR clinical standard resources; and (iii) identification of equivalent data values into SNOMED CT terminology; and (iv) iteration from (i) to (ii) for addition of new variables of interest, enriching the ASCAPE data model in each iteration.

A layer is designed to fully isolate users from transformation process. This layer (i) is transparent for clinical sites and data analysts and (ii) allows users unfamiliar with clinical standards to be able to manage the transformation framework and subsequent data analysis. The layer is supported by an API for users to interact with it, ensuring data entry into the database in a unified way and retrieving data in a common and standardized way.

This process guarantees outcomes of standardized databases, interoperability and resource optimization. Acquisition of new data will adhere to the structure and semantic model described, leading to less errors and a smoother integration within the ASCAPE system. Using a unified and homogenized dataset as data source for AI algorithms, alleviates integration burdens.

### Data analysis

Based on available datasets through ASCAPE, we will utilize standard procedures of AI and ML in processing and analyzing patients’ data collected from different sources to propose personalized predictions and interventions for specific QoL issues. A general workflow regarding the standard ML approach includes three steps:

- Collected data are usually raw and it is necessary to pre-process and curate them by application of adequate algorithms. The aim is to prepare datasets that are as reliable as possible for building ML models.
- Appropriate / adequate algorithms and methods for processing and building models are selected. As different models give results with different reliability and accuracy, it is necessary to identify the predictive models with the best performances. Such models are promising and should further be tested using validation datasets (usually different than these used in building models but with the same structure and closely related to them).
- Trained and validated models are used in clinical practice to support decision-making process.

The first two steps are necessary to build predictive models that are as reliable and robust as possible whereas the third step refers to the implementation and evaluation of previously trained models in clinical practice.

The workflow that is adopted in ASCAPE framework regarding use of datasets and building predictive models has four aspects:

1. Data pre-processing: Available datasets can have missing values for different variables. There are several imputation methods that are appropriate to deal with missing values before training and evaluating ML models. The objectives of this process are to investigate the variables that are more sensitive to missing values, how missing values can influence prediction for individuals, and whether there is an acceptable percentage of missing values to avoid significant influence of prediction quality.
  Missing values are added only within top-ranked features as top-ranked features are more probable to negatively influence prediction performance, when having missing values.
2. Variable selection: This activity is devoted to selecting a subset of relevant features from dataset containing all collected data for patients. In these analyses, most frequently used variable ranking algorithms are applied. After prioritizing variables according to relevant measure, it is necessary to discuss the importance and influential potential of top ranked variables together with clinical experts. Based on consensus, the most appropriate variables should be selected and considered in further activities. In fact, the crucial effect of this phase is identification of the most informative variables for predicting the QoL issues (variables with the highest predictive power for each QoL issue).
3. Predictive models training: The main idea of this stage is to model, design, and optimize ML algorithms that will handle health-related patient data to achieve prediction for each QoL issue / outcome variable. In this stage, intensive experiments using standard algorithms for training classification and regression models are performed. The choice of the trained predictive models is based on several design criteria and metrics: accuracy, privacy-accuracy trade-offs, scalability, etc. Finally, these models are going to be used to process newly collected patients’ data.
  The training models will consider datasets in different periods of patients’ follow up. The idea is to train models to predict QoL issues in the near future for three-months period from baseline to 12 months based on available data.
4. Analytics and real-life evaluation of predictive models. This phase is oriented towards intensive evaluation of the best built models within ASCAPE framework. The essential goal is the adoption and use in clinical practice of the final ML models selected based on all previously conducted steps and experiments. These models are planned to be used further on clinical sites using currently available patients’ data to help doctors in predicting QoL issues for each patient and proposing adequate personalized interventions.

Once the best models are achieved, selected, and put in daily clinical practice, the process is not finished. In fact, ASCAPE framework is a dynamic ecosystem that foresees continuous models’ updates, adjustments and improvements based on newly collected data for existing patients, and new patients in different settings. Local models adopted at local level (data collected from a specific study site) should be constantly re-evaluated based on new local datasets but they will also be adjusted through global models from the ASCAPE cloud. Global models are assembled from all study sites as well as other local Hospitals that will be connected to the ASCAPE system at a later stage. Such predictive models are fine-tuned and condense experiences from the most reliable predictive models. As global models are also continuously updating and adjusting from multiple sources (local and global), they possess high predicting power and may be better choice than locally used models. Healthcare providers will be, therefore, able to implement both local and global models for their patients and select potential interventions for specific QoL issues based on their clinical judgment on which model suggested more logical results and action plans.

### Evaluation process

The evaluation of the AI-based follow-up strategy through the ASCAPE platform will be based on two axes:

A. Patient-centric evaluation

To capture patients’ experience with AI-based follow-up, we will use a mixed methods analysis (initial quantitative approach followed by qualitative methods to interpret the initial quantitative results).

Regarding patients’ experience, the focus of the evaluation will be the experience to be followed with the help of an AI-based system per se, patients’ satisfaction with this type of follow-up, potential barriers and facilitators of using wearables during follow-up, and motivation for following interventions based on AI-based follow-up.

Additional patient-centric evaluation aspects will be:

Patients’ engagement (number of questionnaires submitted per patients; total time that the patients used the wearables)

Measures of effectiveness of AI-based follow-up (patients’ adherence to AI-based proposed intervention; assessment of QoL over time)

B. Doctor-centric evaluation

The doctor-centric evaluation of AI-based follow-up will be focused on three aspects:

1. Impact of AI-based follow-up on relevant metrics in clinical practice. Within this aspect, the following evaluation metrics (gathered as physicians’ views and experience by using AI-based models) will be considered: improvement in patient-doctor relationship; AI-based follow-up’s efficiency to capture relevant QoL issues on time; changes in management or referrals made due to AI-based predictions; usefulness of the information provided by AI-based models; acceptability of integrating AI-based follow-up into clinical practice; assessment of the time needed to use AI-based follow-up in clinical practice.
2. Interaction between AI-based follow-up system and physicians.
  This aspect includes issues related to the interaction between the AI-based follow-up platform and physicians as usability, accessibility, and qualitative assessment of the interface.
3. Experience using the AI-based follow-up platform.

This aspect includes the more general issues on physicians’ experiences in using the AI-based follow-up platform as trustworthiness, how confident physicians are regarding the reliability of AI-based follow-up, and psychological aspects in using an AI-based platform in clinical practice as perceived substitution crisis and behavioural intention.

Considering the complexity of the doctor-centric evaluation process, both quantitative and qualitative approaches will be used to ensure a wide coverage of evaluation metrics.

### Sample size considerations

As the study includes no control group and the study can be considered as a proof-of-concept study on the ability to use AI as a part of follow-up regarding QoL in cancer patients, no formal sample size calculation has been performed. Approximately 500 patients (350 with breast cancer and 150 with prostate cancer) are planned to be included during the enrolment period.

### Ethics and dissemination

The ASCAPE trial ethical considerations are headed towards two main axes: the first lies to the innovative scope of the study, since it is the first to assess AI regarding the two most common types of cancer and the second is related to the protection of personal of cancer patients, as dictated under the applicable EU legal framework, the General Data Protection Regulation (GDPR).

Since this is a non-invasive study without potential direct physical harm from the proposed interventions (the proposed interventions will be based on current clinical practice and will be implemented only after shared decision-making), namely the utilization of AI-based methods for follow-up of health-related QoL in patients with breast- or prostate cancer, the first axis does not pose a vital threat to the study.

Concerning GDPR, ASCAPE is committed to high standards of information security, privacy and transparency and place a high priority on protecting and managing data in accordance with ethical standards and data protection legislation, both at European and national level. In essence, ASCAPE architecture has designed in accordance with the requirements set under the GDPR.

Within the context of the ASCAPE project, the processing of personal data envisioned will be performed in accordance with the key principles and that should underly such processing (Article 5 of the GDPR), as well as other applicable provisions of the GDPR, including the following:

- Personal data will be solely processed for clearly pre-defined purposes.
- Personal data will be adequate, relevant, and limited to what is necessary in relation to the purposes for which they are processed (data minimization).
- Personal data shall be kept accurate and up to date.
- Personal data will be retained as long as it is necessary for the accomplishment of the predefined purpose(s).
- Personal data will be kept secure through the implementation of appropriate technical and organizational measures (integrity and confidentiality).
- Personal data will not be transferred to countries outside the EU that do not afford the necessary guarantees for an adequate level of protection.

Note that all processing of personal data to be conducted in the context of ASCAPE project will be performed based on a consent provided by participants, prior to the launch of any processing activities relating to their data. Participants will be, thus, properly informed in a clear language on the purpose of ASCAPE research, on the types of their personal data that will be collected and further processed, as well as on the respective rights they are entitled to. Participants maintain their right to withdraw their originally given consent at any time, following a well-formed and easily accessible withdrawal procedure provided by ASCAPE.

### Patient and public involvement

No patient involved in the design of the study. Patients will be involved in the evaluation of the ASCAPE-based strategy as it is described on “Patient-centric evaluation”.

## Discussion

ASCAPE is the first research project that will prospectively investigate an AI-based approach, towards an individualized follow-up strategy for patients with breast- or prostate cancer focusing on patients’ QoL issues. The main aim of the project is to offer substantial benefits for after-treatment health-related QoL improvements.

The ASCAPE platform will be tested into different settings including large tertiary hospitals, primary care setting, and a setting supporting patients remotely, in parallel with their existing healthcare professionals. In addition, the study sites will utilize different data collection and patient interaction modalities using technological means, including a specialized mobile application, a wearable device, websites, and e-mails. They will also demonstrate different levels of integration with ASCAPE platform: two sites will only modify their IT-systems to provide ASCAPE with patient data, thus having to rely on the ASCAPE Dashboard to offer AI-based prediction models to healthcare professionals, whereas the remaining two will attempt to provide full integration of ASCAPE into current IT-system.

The different approaches on several clinical and technical aspects among the four study sites of the ASCAPE project pose significant challenges in the preparation and execution of the study. However, this heterogeneity among study sites reflects the heterogeneity among different clinical settings where ASCAPE platform will be able to be implemented in the future. As a result, the results of the current study can ensure the generalizability of the ASCAPE platform is different settings.

To further serve the scope of the generalizability of the current study results, we have designed a comprehensive evaluation framework, covering doctor- and patient-centric evaluation (described in the current protocol) but also AI-centric evaluation of the algorithms and health economics-centric evaluation as additional parts of the evaluation process.

It is important to highlight that the ASCAPE platform is designed to be a practical tool for healthcare professionals, who will be responsible for determining how to use the provided information to their patients’ follow-up strategy. AI is not mature enough to suggest interventions directly to the patient without doctor’s medical opinion. For this reason, ASCAPE’s main users are healthcare professionals, and its focus is to provide concise and user-friendly presentation of results in accordance to their requirements. Patients will interact with ASCAPE indirectly through providing information that will help ASCAPE provide doctors more accurate AI-based predictions and more appropriate intervention recommendations.

The ASCAPE project will build and evaluate an AI-based platform that represents a paradigm shift regarding the follow-up approach for cancer patients focusing on improving their QoL after cancer treatment, which is an unmet need for cancer survivors (4–7). The ASCAPE project also represents a paradigm shift on how to use Big Data in cancer care through democratizing the knowledge and the access to AI and Big Data related innovations. In fact, ASCAPE aims to minimize the gap between large- and small-sized healthcare providers by making the ASCAPE AI-based tools available to any health care provider that could utilize them for the benefit of cancer patients around Europe and the world.

## Supporting information

S1 Table Proposed interventions for breast cancer patients. (DOCX)

S2 Table Proposed interventions for prostate cancer patients. (DOCX)

S1 File (PDF)

S2 File (DOCX)

S3 File (DOCX)

S4 File (PDF)

S5 File (DOCX)

S6 File (DOCX)

S7 File (DOC)

## Abbreviations

AI: Artificial Intelligence
API: Application Programming Interface
BHC: Barcelona Hospital Clinic
DASH: Disability of Arm, Shoulder and Hand questionnaire
EORTC: European Organization for Research and Treatment of Cancer
GAD7: General Anxiety Disorder 7-item
GDPR: General Data Protection Regulation
HADS: Hospital Anxiety and Depression Scale
HIS: Hospital Information System
HL7 FHIR: Fast Healthcare Interoperability Resources
IIEF-5: International Index of Erectile Function
ML: Machine Learning
NKUA: National and Kapodistrian University of Athens
PCCs: Primary Care Centers
PHQ9: Patient Health Questionnaire
QoL: Quality of Life
ORB/Uppsala: Örebro and Uppsala University Hospitals
