# Supplementary material for: Artificial intelligence supporting cancer patients across Europe—The ASCAPE project
Source: PLoS One. 2022 Apr 21;17(4):e0265127. doi: 10.1371/journal.pone.0265127 (PMC9022843; doi:10.1371/journal.pone.0265127)
Supplement: S1 Table — (DOCX) [file pone.0265127.s001.docx]

*Supplementary Table 1. Proposed interventions for breast cancer patients*

| **QoL issues breast cancer interventions** | |
| --- | --- |
| **Anxiety** | Anti-stress techniques: mindfulness, Yoga  Movement-based relaxation techniques  Physical activity*  Promotion of positive familiar and social relationships  Psychological support  Psychiatric support  Anxiolytics  Anti-depressants |
| **Depression** | Anti-stress techniques: mindfulness, Yoga  Movement-based relaxation techniques  Physical activity*  Promotion of positive familiar and social relationships  Psychological support  Psychiatric support  Anti-depressants |
| **Fatigue** | Anti-stress techniques: mindfulness, Yoga  Movement-based relaxation techniques  Physical activity*  Promotion of positive familiar and social relationships  Psychological support  Nutrition consultation  Interventions for sleep disturbances |
| **Weight gain** | Diet  Nutrition consultation  Physical activity* |
| **Hot flushes** | Movement-based relaxation techniques  Physical activity*  Psychological support  Anti-depressants  Anticonvulsant agents |
| **Joint pain** | Movement-based relaxation techniques  Physical activity*  Acupuncture  Switch from aromatase inhibitor  Switch aromatase inhibitor to tamoxifen  Analgesic (NSAID or others)  Duloxetine |
| **Neurotoxicity** | Movement-based relaxation techniques  Physical activity *  Acupuncture  Antidepressants |

*Abbreviations:* QoL, quality of life; NSAID: non-steroidal anti-inflammatory drugs.

** Physical activity refers to moderate physical activity for a total of 150 minutes weekly (eg 5 days of 30 minutes exercise). Moderate exercise refers to 3-4 metabolic equivalents (MET) and can be for example cycling at road with a speed of 16 km/hour or at a trainer at 50 watts, walking at a pace of 4.8-5 km/hour, gardening, and light weight lifting*
