# Supplementary material for: Artificial intelligence supporting cancer patients across Europe—The ASCAPE project
Source: PLoS One. 2022 Apr 21;17(4):e0265127. doi: 10.1371/journal.pone.0265127 (PMC9022843; doi:10.1371/journal.pone.0265127)
Supplement: S2 Table — (DOCX) [file pone.0265127.s002.docx]

Supplementary Table 2. Proposed interventions for prostate cancer patients

| **QoL issues prostate cancer interventions** | |
| --- | --- |
| **Anxiety** | Anti-stress techniques: mindfulness, Yoga  Movement-based relaxation techniques  Physical activity *  Promotion of positive familiar and social relationships  Psychological support  Psychiatric support  Anxiolytics  Anti-depressants |
| **Depression** | Anti-stress techniques: mindfulness, Yoga  Movement-based relaxation techniques  Physical activity*  Promotion of positive familiar and social relationships  Psychological support  Psychiatric support  Anti-depressants |
| **Fatigue** | Anti-stress techniques: mindfulness, Yoga  Movement-based relaxation techniques  Physical activity*  Promotion of positive familiar and social relationships  Psychological support  Nutrition consultation  Interventions for sleep disturbances |
| **Weight changes** | Diet  Nutrition consultation  Physical activity* |
| **Hot flushes** | Movement-based relaxation techniques  Physical activity*  Psychological support  Progesterone / progestin  Cyproterone  Anti-depressants  Anticonvulsant agents  Androgen deprivation treatment manipulation |
| **Incontinence** | Conservative treatment:   - pelvic muscle training with or without biofeedback - electrical stimulation - extracorporeal magnetic innervation - compression devices (penile clamps) - lifestyle changes   Surgical management   - slings and artificial urinary sphincters |
| **Sexual dysfunction** | Couple counselling  5DE inhibitors daily or on demand  Intracavernosal injections  Intermittent ADT instead of continuous ADT  Penile prosthesis or pumps |

*Abbreviations:* QoL, quality of life; 5DE, Phosphodiesterase-5; ADT, androgen deprivation therapy.

** Physical activity refers to moderate physical activity for a total of 150 minutes weekly (eg 5 days of 30 minutes exercise). Moderate exercise refers to 3-4 metabolic equivalents (MET) and can be for example cycling at road with a speed of 16 km/hour or at a trainer at 50 watts, walking at a pace of 4.8-5 km/hour, gardening, and light weight lifting*
