# Supplementary material for: Artificial intelligence supporting cancer patients across Europe—The ASCAPE project
Source: PLoS One. 2022 Apr 21;17(4):e0265127. doi: 10.1371/journal.pone.0265127 (PMC9022843; doi:10.1371/journal.pone.0265127)
Supplement: S1 File — (PDF) [file pone.0265127.s003.pdf]

STUDY PROTOCOL

Artificial intelligence Supporting CAncer Patients across Europe

ASCAPE

Version number:	1.1
Date:	2021-11-15

Grant Agreement No	875351
Instrument	Research and Innovation action
Call / Topic	H2020-SC1-DTH-2019 /<br>Big data and Artificial Intelligence for monitoring health status and quality of life after the cancer treatment
Principal Investigator in Sweden	Antonios Valachis

Table of contents

Signature page ... 4
Contact information ... 5
1. Synopsis ... 6
2. Background and rationale ... 8
3. Study objectives ... 8
3.1. Primary objective ... 8
3.2. Secondary objective(s) ... 9
4. Study design and procedures ... 9
4.1. Overall study design ... 9
4.2. Procedures and flow chart ... 9
4.3. End of Study ... 11
5. Subject selection ... 11
5.1. Inclusion criteria ... 11
5.2. Exclusion criteria ... 11
5.3. Screening ... 12
5.4. Withdrawal criteria ... 12
6. Study procedures and data collection ... 12
6.1. Study procedure ... 12
6.2. Quality of life issues and measures ... 13
6.3 QoL issues and proposed interventions ... 14
7. Evaluation process ... 14
7.1. Patient-centric evaluation ... 14
7.2. Doctor-centric evaluation ... 15
8. Statistics ... 15
8.1. Analysis population ... **Error! Bookmark not defined.**
8.2. Statistical analyses ... 15
8.2.1. Statistical methods ... **Error! Bookmark not defined.**
8.3. Sample size calculations ... 16
9. Ethics ... 16
9.1. Compliance to the protocol, GCP and regulations ... 16
9.2. Ethical review of the study ... 16

9.3. Procedure for obtaining informed consent 16
9.4. Data protection 17
9.5. Insurances 18
10. Substantial changes to the study 18
11. Collection, handling, and archiving data 18
11.1. Case Report Form (Forskningspersonsformulär) 18
12. Notification of study completion, reporting, and publication 18
13. References 19
14. Appendix 21

Signature page

Sponsor

I am responsible for ensuring that this protocol includes all essential information to be able to conduct this study. I will submit the protocol and all other important study-related information to the responsible investigator(s) so that they can conduct the study correctly. I am aware that it is my responsibility to hold the staff members who work with this study informed and trained.

Sponsor's signature Date

Antonios Valachis

Coordinating Investigator / Principal Investigator

I have read this protocol and agree that it includes all essential information to be able to conduct the study. By signing my name below, I agree to conduct the study in compliance with this protocol, the Declaration of Helsinki, ICH GCP (Good Clinical Practice) guidelines and the national and international regulations governing the conduct of this clinical study.

I will submit this protocol and all other important study-related information to the staff members and responsible investigators who participate in this study, so that they can conduct the study correctly. I am aware of my responsibility to continuously keep the staff members and responsible investigators who work with this study informed and trained.

I am aware that quality control of this study will be performed in the form of monitoring, possibly audit, and possibly inspection.

Coordinating Investigator / Principal Investigator's signature Date

Antonios Valachis

Contact information

**Role**	
Sponsor	Antonios Valachis<br><br>Department of Oncology, Örebro University Hospital, Sweden<br><br>Tel. 0046735617691<br><br>e-mail: antonios.valachis@oru.se
Coordinating Investigator / Principal Investigator	Antonios Valachis<br><br>Department of Oncology, Örebro University Hospital, Sweden<br><br>Tel. 0046735617691<br><br>e-mail: antonios.valachis@oru.se
Principal investigator (Uppsala)	Henrik Lindman<br><br>Department of Oncology, Akademiska University Hospital, Sweden<br><br>Anthoula Koliadi<br><br>Department of Oncology, Akademiska University Hospital, Sweden
Study nurse (Örebro)<br><br>Study nurse (Uppsala)	

1. Synopsis

Title:	Artificial intelligence Supporting CAncer Patients across Europe – The ASCAPE project in Sweden
Rationale/Purpose:	Many cancer patients experience adverse effects of cancer or treatment, which can considerably decrease quality of life (QoL). The current strategy of supporting cancer patients/survivors does not meet their needs due to the limited personalized-based approach in rehabilitation plan and the lack of healthcare, financial and other resources.<br><br>ASCAPE (Artificial intelligence Supporting CAncer Patients across Europe) is a collaborative research project involving 15 partners from 7 countries, including academic medical centers, SMEs (small and medium-sized enterprises), research centers and universities, aiming to leverage the recent advances in Big Data and AI (Artificial Intelligence) to support cancer patients' QoL and health status. Specifically, ASCAPE aims to provide personalized- and AI-based predictions for QoL issues in breast- and prostate cancer patients as well as suggest potential interventions to their physicians.
Study objectives:	To evaluate the QoL aspects in patients with breast and prostate cancer treatment with curative intention during the 1st year of diagnosis and develop and implement Artificial Intelligence (AI)-based models predicting potential impairment in QoL using active monitoring data.<br>Secondary aims are to investigate the clinicians´ views and experiences in using AI-based models in clinical practice and patients´ experiences in the concept of AI-based follow-up
Study design:	Multicentric, non-interventional, prospective longitudinal study.
Study population:	The study will include 2 cohorts:<br>Breast cancer cohort: Patients with early breast cancer planned for curative treatment with surgery with or without oncological treatment.<br>Prostate cancer cohort: Patients with localized prostate cancer planned for curative treatment with surgery and/or radiotherapy.
Number of subjects:	As the study does not include a control arm, no formal sample size calculation has been performed. Considering the number of newly diagnosed breast cancer and prostate cancer each year and the expected inclusion rate, approximately 120 breast cancer patients

	and 80 prostate cancer patients are expected to be included during the inclusion period.
Inclusion criteria:	For breast cancer cohort:<br><br>1. Histologically proven breast cancer (any subtype).<br>2. No clinical evidence of metastatic disease.<br>3. Able for curative treatment with surgery with or without oncological treatment (endocrine therapy, chemotherapy, radiotherapy as neoadjuvant or adjuvant).<br>4. No prior malignant tumor during the previous 5 years, except for in situ carcinomas of the cervix or basal or squamous cell carcinomas of the skin adequately treated.<br>5. Signed informed consent before study entry.<br>6. Ability to utilize smartphone, apps, and wearables.<br><br>For prostate cancer cohort:<br><br>1. Histologically proven prostate cancer<br>2. No clinical evidence of metastatic disease.<br>3. Able for curative treatment with radiotherapy irrespectively the type of radiotherapy (external, brachytherapy, combination). Patients with postoperative radiotherapy will also be included.<br>4. No prior malignant tumor during the previous 5 years, except for basal or squamous cell carcinomas of the skin adequately treated.<br>5. Signed informed consent before study entry.<br>6. Ability to utilize smartphone, apps, and wearables.
Exclusion criteria:	For both cohorts:<br><br>1. Patients with metastatic disease not able for curative treatment.<br>2. Patients with known history of allergy to the wearable material.<br>3. Inability to give informed consent.<br>4. Inability/ no access to use smartphones, applications, wearable devices or internet services.
Study period:	Q2 2021 – Q4 2022

2. Background and rationale

The number of cancer survivors is steadily increased over time due to the advances in cancer diagnosis and treatment strategies (1). Considering the high incidence of breast- and prostate cancer in women and men, respectively and the increased survival for these malignancies over time, it is not surprising that the most prevalent cancer types among cancer survivors is breast cancer for women and prostate cancer for men (1).

Many cancer patients experience adverse effects of cancer or treatment, which can considerably decrease quality of life (QoL) (2,3). The current strategy of supporting cancer patients/survivors does not meet their needs due to the limited personalized-based approach in rehabilitation plan and the lack of healthcare, financial and other resources (4-7).

ASCAPE (Artificial intelligence Supporting CAncer Patients across Europe) is a collaborative research project involving 15 partners from 7 countries, including academic medical centers, SMEs (small and medium-sized enterprises), research centers and universities, aiming to leverage the recent advances in Big Data and AI (Artificial Intelligence) to support cancer patients' QoL and health status. Specifically, ASCAPE aims to provide personalized- and AI-based predictions for QoL issues in breast cancer patients as well as suggest potential interventions to their physicians.

During the first part of the project, large-scale retrospective datasets with breast or prostate cancer patients will be analyzed to develop and train AI-based models for specific QoL issues. During the second part of the project, that is described in the current protocol, a multicenter prospective longitudinal study is planned. Eligible patients will be followed for one year with validated questionnaires regarding different QoL issues, and wearables that will collect active monitoring data on physical activity, sleep pattern, and heart rate.

The collected data will be used to further train and optimize the AI-based models and personalized-based intervention suggestions.

Based on the retrospective and prospective data, an ASCAPE-integrated prototype will be developed, enabling personalized- and AI-based predictions and intervention suggestions.

This approach will be evaluated at the end of the prospective study regarding patients´ and physicians´ experience (with a mixed methods approach), as well as health economics.

3. Study objectives

3.1. Primary objective

The Örebro pilot has two aims: the primary aim is to evaluate the QoL aspects in patients with breast and prostate cancer treatment with curative intention during the 1st year of diagnosis and to facilitate, through structured data collection, the development of AI-based models predicting potential impairment in QoL aspects through the ASCAPE.

3.2. Secondary objective(s)

The secondary aims of the pilot are to implement the ASCAPE-based platform for predictions and interventions in a real-world clinical setting and investigate the clinicians' views and experiences in using AI-based models in clinical practice and patients' experiences in the concept of AI-based follow-up.

4. Study design and procedures

4.1. Overall study design

The study is a multicenter single-arm, longitudinal cohort study including patients with breast or prostate cancer treated with curative intention.

Patients with early breast cancer planned for curative treatment with surgery with or without oncological treatment at the Örebro University Hospital or Uppsala University Hospital, respectively will be eligible for study inclusion. Besides, patients with localised prostate cancer planned for curative treatment with radiotherapy at the Örebro University Hospital will be eligible for study inclusion.

4.2. Procedures and flow chart

All patients meeting the inclusion criteria will be asked to be included to this longitudinal study. All patients will be treated according to the clinical practice.

Upon inclusion to the study, the patients will have an additional follow-up plan that consists of self-reported electronic QoL-questionnaires (at baseline and every three months during one year), use of wearables (Fitbit Inspire HR®, provided by the study) during one year, and follow-up contact (either through telephone or in-person visits) with the treating oncologist or study nurse every three months during one year.

Information collected through questionnaires and wearables will be used to develop AI-based models for prediction of impairment in specific QoL-issues. The AI-based models will be continuously trained by provided information and will also be used to provide individual-based predictions for each patient.

During the protocol-specified follow-up contacts, the healthcare provider will have access to patient's responses to questionnaires and individual-based predictions based on the AI-models. The predictive models will inform the healthcare provider on which QoL-issues are predicted to be impaired in the near future for the patient and will also provide suggestions for interventions to prevent or improve the impaired QoL issues. The healthcare provider is responsible to discuss the AI-based prediction with the patient and decide, in a shared decision-making manner, the most suitable intervention for the patient that might or might not be the same as the one suggested from the AI-based model. The chosen intervention should, therefore, be based on evidence-based medicine and clinical experience and not solely on the AI-based suggestion.

The effectiveness of the proposed intervention to the specific QoL issue will be followed through a short electronic questionnaire that will be sent to the patient after a time period that

will be decided by the healthcare provider. Changes in the proposed intervention or the effectiveness of a new proposed intervention will also be followed by the same way.

Each patient will be followed using the aforementioned follow-up plan for one year. At the end of study period, the patients will be answer to a questionnaire evaluating their experience and satisfaction with the AI-based follow-up, their experience in using wearables in a daily basis, and potential barriers or facilitators for this type of follow-up. Some patients will also be part in focused interviews to provide qualitative aspects on the evaluation.

The flowchart of study procedure is shown in table 1.

Table 1. Flowchart of study procedure

Procedure	Screening Inclusion visit	Visit 1<br>Baseline	Visit 2<br>3 months<br>(+/- 14 days)	Visit 3<br>6 months<br>(+/- 14 days)	Visit 4<br>9 months<br>(+/- 14 days)	Visit 5<br>12 months<br>(+/- 14 days)
Inclusion and exclusion criteria	x					
Informed consent		X				
Medical history/ concomitant medications		X				
Provide the wearable		X				
QoL-questionnaires		X	x*	x*	x*	x*
In-person contact with healthcare provider	x	X				
In-person or telephone contact with healthcare provider			X	x	x	x
Questionnaire on the effectiveness of a specific intervention****			X	x	x	x
Evaluation-questionnaire						x**
Interview for qualitative evaluation						x***

*The QoL-questionnaires will be sent 14 days before scheduled contact

**The evaluation questionnaire will be filled out after the 5th contact

***The interview for qualitative evaluation will be scheduled at the 5th contact but it will be performed after the 5th contact

****The evaluation of the effectiveness of a specific intervention can be electronically sent at any time during the study period. The time will be decided by the treating oncologist.

4.3. End of Study

The study ends when the last study participant has completed the last follow-up including the evaluation part.

5. Subject selection

5.1. Inclusion criteria

The inclusion criteria for breast cancer patients are:

1. Histologically proven breast cancer (any subtype).
2. No clinical evidence of metastatic disease.
3. Able for curative treatment with surgery with or without oncological treatment (endocrine therapy, chemotherapy, radiotherapy as neoadjuvant or adjuvant).
4. No prior malignant tumor during the previous 5 years, except for in situ carcinomas of the cervix or basal or squamous cell carcinomas of the skin adequately treated.
5. Signed informed consent before study entry.
6. Ability to utilize smartphone, apps, and wearables.

The inclusion criteria for prostate cancer patients are:

1. Histologically proven prostate cancer
2. No clinical evidence of metastatic disease.
3. Able for curative treatment with radiotherapy irrespectively the type of radiotherapy (external, brachytherapy, combination). Patients with postoperative radiotherapy will also be included.
4. No prior malignant tumour during the previous 5 years, except for basal or squamous cell carcinomas of the skin adequately treated.
5. Signed informed consent before study entry.
6. Ability to utilize smartphone, apps, and wearables.

5.2. Exclusion criteria

The exclusion criteria for both breast and prostate cancer patients are:

1. Patients with metastatic disease not able for curative treatment.
2. Patients with known history of allergy to the wearable material.
3. Inability to give informed consent.
4. Inability/ no access to use smartphones, applications, wearable devices or internet services.

5.3. Screening

All newly diagnosed breast or prostate cancer patients are discussed on the weekly dedicated multidisciplinary team meeting. All breast cancer patients are, thereafter, referred to the Department of Oncology either before (when preoperative treatment is recommended or in case of primary metastatic disease) or after surgery. Similarly, all prostate cancer patients eligible for curative radiotherapy are referred to the Department of Oncology.

The follow-up of breast cancer patients after treatment in both the Uppsala and Örebro regions is performed by the Departments of Oncology, mainly through dedicated oncology nurses. Besides, the follow-up of prostate cancer patients after curative radiotherapy in Örebro region is also performed by the Department of Oncology. ASCAPE will, therefore, be integrated into the current follow-up practice of breast and prostate cancer patients as an additional tool for oncologists and oncology nurses.

All potentially eligible patients for the study will be identified through the multidisciplinary team meetings. The patients will be informed about the possibility to participate to the study at their first visit at the Departments of Oncology in Uppsala or Örebro University Hospitals for breast cancer and at the Department of Oncology in Örebro University Hospital for prostate cancer.

5.4. Withdrawal criteria

Subjects can discontinue their participation in the study at any time without any consequence to his/her continued treatment and follow-up. The investigator/sponsor can at any time terminate the study for a subject due to, e.g the subject does not follow procedures in the study protocol.

If the subject discontinues the study, follow-up of this subject will be performed according to the clinical routine.

6. Study procedures and data collection

6.1. Study procedure

All potentially eligible patients for the study will be identified through the multidisciplinary team meetings. The patients will be informed about the possibility to participate to the study at their first visit at the Department of Oncology in Örebro University Hospital (breast- and prostate cancer patients) or Uppsala University Hospital (breast cancer patients). Informed consent will be obtained.

At baseline (after informed consent), the following data will be collected:

- patients' characteristics
- tumour characteristics
- treatment-related information
- questionnaire about lifestyle (Appendix 1a and 1b) and nutrition (Appendix 2)
- baseline QoL measurements (detailed description in section 6.2).

Each included patient will also receive a wearable (Fitbit Inspire HR®) with ability for continuous measurements of heart rate, activity parameters, and sleep parameters. Each patient will have the wearable during the whole study period of 12 months.

The follow-up will be performed every 3 months using QoL-questionnaires before each contact for up to 12 months. An oncologist will be responsible for the follow-up with possibility to delegate the responsibility to a dedicated oncology nurse. The following aspects should be covered at each contact:

- Patient's QoL and potential QoL issues
- Individualized prediction of QoL based on AI-based models (when this function is operational)
- Potential interventions for improvement of QoL including interventions proposed by AI-based models
- Planning on when the effectiveness of the proposed intervention will be evaluated

During the study follow-up (12 months for each patient), any intervention aimed to improve a measured quality of life aspect will be captured and additional follow-up (within 1 to 4 weeks depending on the nature of intervention) can be scheduled at the oncologist discretion.

6.2. Quality of life issues and measures

The study will focus on 15 QoL issues for breast cancer and 12 for prostate cancer. The identification of QoL issues to be predicted within ASCAPE for breast and prostate cancer was a multistep procedure that was based on current evidence and clinical expertise.

The 15 QoL issues for breast cancer to be predicted through AI-based models are: anxiety, body changes, body image, cognitive impairment, depression, dry vagina, emotional symptoms (loneliness), fatigue, hot flushes, insomnia, joint pain, local symptoms after surgery, lymphedema, neurotoxicity, and sexual dysfunction.

The 12 QoL issues for prostate cancer to be predicted through AI-based models are: anxiety, bowel dysfunction, cognitive impairment, depression, erectile dysfunction, fatigue, hot flushes, incontinence, low urinary tract symptoms, loss of libido, musculoskeletal pain, and weight changes.

The following questionnaires will be used to capture different aspects on breast cancer patients' QoL:

- BREAST-Q (mastectomy or breast conserving therapy)(8) at baseline (Appendix 3a and 3b) followed by BREAST-Q postoperative (Appendix 3a and 3b)
- EORTC QLQ C30 (9): at baseline and every three months (Appendix 4)
- EORTC QLQ-BR23 (10): at baseline and every three months (Appendix 5)
- Hospital Anxiety and Depression Scale (HADS)(11): at baseline and every three months (Appendix 6)
- Disability of Arm, Shoulder and Hand questionnaire (DASH)(12): every three months (Appendix 7)

The following questionnaires will be used to capture different aspects on prostate cancer patients' QoL:

- EORTC QLQ C30 (9): at baseline and every 3 months (Appendix 4)
- EORTC QLQ-PR25 (13): at baseline and every 3 months (Appendix 8)
- Hospital Anxiety and Depression Scale (HADS) (11): at baseline and every three Months (Appendix 6)
- International Index of Erectile Function (IIEF) (14): at baseline and every three Months (Appendix 9)

All questionnaires are extensively validated and are recommended by the Breast Cancer Evaluation Database to Guide Effectiveness (EDGE) Task Force and the Prostate Cancer EDGE Task Force, respectively for the clinical measures of health-related QoL (15-18).

6.3 QoL issues and proposed interventions

The identification process for suitable QoL issues for proposing interventions through the AI-based models was a multistep procedure, similar to the process for identifying QoL issues for predictions.

Seven breast cancer QoL issues are suitable for proposing interventions through AI-based models including anxiety, depression, joint pain, fatigue, neurotoxicity, hot flushes, and weight gain.

Seven prostate cancer QoL issues are suitable for proposing interventions though AI-based models including anxiety, depression, fatigue, incontinence, weight changes, sexual dysfunction, and hot flushes.

A list about the potential interventions that will be proposed through AI-based models for each QoL issue is presented in Appendix 10. The list of potential interventions is based on current evidence and clinical guidelines.

7. Evaluation process

7.1. Patient-centric evaluation

To capture patients' experience with AI-based follow-up, we will use a mixed methods analysis (initial quantitative approach followed by qualitative methods to interpret the initial quantitative results).

Regarding patients' experience, the focus of the evaluation will be the experience to be followed with the help of an AI-based system per se, patients' satisfaction with this type of follow-up, potential barriers and facilitators of using wearables during follow-up, and motivation for following interventions based on AI-based follow-up.

Additional patient-centric evaluation aspects will be:

Patients' engagement (number of questionnaires submitted per patients; total time that the patients used the wearables)

Measures of effectiveness of AI-based follow-up (patients' adherence to AI-based proposed intervention; assessment of QoL over time)

7.2. Doctor-centric evaluation

The doctor-centric evaluation of AI-based follow-up will be focused on three axes:

1. Impact of AI-based follow-up on relevant metrics in clinical practice. Within this aspect, the following evaluation metrics (gathered as physicians' views and experience by using AI-based models) will be considered: improvement in patient-doctor relationship; AI-based follow-up's efficiency to capture relevant QoL issues on time; changes in management or referrals made due to AI-based predictions; usefulness of the information provided by AI-based models; acceptability of integrating AI-based follow-up into clinical practice; assessment of the time needed to use AI-based follow-up in clinical practice.

2. Interaction between AI-based follow-up system and physicians

This aspect includes issues related to the interaction between the AI-based follow-up platform and physicians as usability, accessibility, and qualitative assessment of the interface.

3. Experience using the AI-based follow-up platform

This aspect includes the more general issues on physicians' experiences in using the AI-based follow-up platform as trustworthiness, how confident physicians are regarding the reliability of AI-based follow-up, and psychological aspects in using an AI-based platform in clinical practice as perceived substitution crisis and behavioural intention.

Considering the complexity of the doctor-centric evaluation process, both quantitative and qualitative approaches will be used to ensure a wide coverage of evaluation metrics.

8. Statistics

8.1. Statistical analyses

For discrete QoL issues, we will examine the efficiency of classification-based machine learning models trained using decision tree learning algorithms, nearest-neighbors based algorithms, probabilistic learning algorithms, support vector machines and (deep) neural networks. Regressive counterparts of aforementioned methods will be analyzed for numeric QoL outcome variables including also regression specific methods (e.g., ridge regression, lasso regression and elastic net regression). The accuracy of trained models will be estimated relying on standard machine learning validation procedures such as the K-fold cross-validation and leave-one-out cross-validation. Additionally, a primary aim of the project is to investigate the accuracy of collectively trained predictive QoL models in federated learning settings in which training datasets are not exchanged between partners involved in the model training process.

The AI-based follow-up platform will utilize state-of-the-art explainability techniques to make the machine learning models' predictions transparent and comprehensible for the patient and the physician.

The evaluation part of the study will use descriptive statistics to summarize and present the data.

8.2. Sample size calculations

As the study does not include a control arm, no formal sample size calculation has been performed. Considering the catchment area of the two healthcare regions (Uppsala Region län and Örebro Region län), the number of newly diagnosed breast and prostate cancer each year, and the expected inclusion rate, approximately 120 women with breast cancer and 60 men with prostate cancer, respectively, are expected to be included.

9. Ethics

9.1. Compliance to the protocol, GCP and regulations

The study will be performed in accordance with the study protocol, ICH-GCP E6 (R2) and all applicable legal and ethical requirements, including the revised Declaration of Helsinki on Ethical Principles for Medical Research Involving Human Subjects[1] adopted by the World Medical Association and the General Data Protection Regulation[2] (GDPR). This is to ensure the fundamental rights of the study subjects, such as safety and privacy, as well as the quality of the data collected.

9.2. Ethical review of the study

The final study protocol, including the final versions of the informed consent forms and other information provided to subjects, must first be approved by the Swedish Ethical Review Authority (Etikprövningsmyndigheten, EPM). The EPM must be informed of any changes in the study protocol in accordance with the applicable requirements within Örebro University Hospital and Akademiska University Hospital.

9.3. Procedure for obtaining informed consent

The principal investigator at each site shall ensure that the subject is given full and adequate oral and written information in Swedish about the study, its purpose, any risks and benefits as well as inclusion and exclusion criteria. Subjects will also be informed that they are free to withdraw their participation in the study at any time without having to provide a reason and without this withdrawal entailing any consequences whatsoever for any treatment they are receiving at the moment of the potential withdrawal or any future treatment Subjects will be given the opportunity to ask questions and be allowed time to consider the provided information. If the person chooses to participate, both the subject and the investigator shall sign the informed consent form in Swedish, which shall subsequently allow their involvement

---

[1] WMA Declaration of Helsinki – Ethical principles for medical research involving human subjects adopte d on the 18th July 2018, available at:https://www.wma.net/policies-post/wma-declaration-of-helsinki-ethical-principles-for-medical-research-involving-human-subjects/

[2] Regulation (EU) 2016/679 of the European Parliament and of the Council of 27 April 2016 on the protection of natural persons with regard to the processing of personal data and on the free movement of such data, and repealing Directive 95/46/EC (General Data Protection Regulation) (Text with EEA relevance)

in the study A copy of the subject information form as well as the informed consent form itself shall be provided to the subject. The subject’s signed and dated informed consent must be obtained before performing any study-specific activity in the study. If, however, new information is added to the study, the subject has the right to reconsider whether he/she will continue their participation. Note that all relevant procedures are detailed under the dedicated ASCAPE report "D7.1 – Human - Requirement No. 2". The report elaborates -among other- on the procedures for the recruitment of research participants, including with respect to the acquisition of consent for the participation to the study. The report has been submitted to the European Commission[3].

9.4. Personal data protection

The personal data processing throughout the study will be performed in accordance with the provisions of the General Data Protection Regulation (GDPR) and other applicable legislation. In line with the earlier stated applicable personal data protection framework providing for the principle of data minimization, for instance, each subject who participates in the study will be identified by a subject number on a subject identification list; in particular, subjects who participate in the study are coded with a specific study identification number. All subjects are registered in a subject identification list (subject enrolment and identification list) that connects the subject’s name and personal number with a study identification number. Notably, since the personal data will be analyzed within the study consortium by partners outside Sweden-- in addition to the relevant provisions under the Grant Agreement and the consortium agreement-appropriate contractual arrangements in place provide additional guarantees for compliance with the above-mentioned regulations.

As far as the right to data protection is further concerned, except for the informed consent form template mentioned above, the research participants will be presented with all necessary information in Swedish regarding the processing of their personal data, including information on the exact types of personal data to be collected and the specific purpose to be fulfilled. Subsequently, they will be requested to express their consent in line with the requirements of the GDPR, that dictate -among other- a clear affirmative action, such as a signature. The acquisition of a valid consent will provide the legal basis for the processing of personal data for the purposes of ASCAPE[4].

Moreover, the concrete technical and organizational measures in place by ASCAPE consortium partners to ensure protection of personal data of research participants is elaborated extensively under the report "D7.2 POPD - Requirement No. 5", which is formally approved by European Commission. In terms of technical measures, for instance, homomorphic encryption will be applied throughout the project to encrypt personal data. As far as organizational measures are concerned, ASCAPE consortium provides -among other- for the function of a Data Protection Officer (DPO).

[3] This report is confidential. It accessible only to ASCAPE Consortium partners and the European Commission.

[4] Note that a relevant discussion , including the relevant consent templates is included under the previously mentioned: "D7.1 – Human - Requirement No. 2

9.5. Insurances

All participants are insured through the Swedish patient insurance (Patientskadeförsäkring). No further insurance is applicable for the project.

10. Substantial changes to the study

Substantial changes to the signed study protocol are only possible through approved protocol amendments and by agreement from all responsible persons. Information on non-substantial changes should be clearly noted in the amended protocol.

In the event that substantial changes to the protocol (e.g., changing of the main objective, methods to measure the outcomes) will be made during the course of the study, approval from the Swedish Ethical Review Authority (Etikprövningsmyndigheten, EPM) shall be obtained before any changes are implemented. A change that concerns a new site, new investigator or a new study patient information sheet shall also be approved by EPM.

Non-substantial changes will be recorded and later entered in documentation that is submitted, for example in any subsequent notifications of a substantial change or in connection with End of Trial reporting.

11. Collection, handling, and archiving data

All data, including both personal and non- personal data, will be registered, managed, and stored in a manner that enables correct reporting, interpretation, and verification in accordance with the applicable rules and policies in place.

11.1. Electronic Case Report Form (Forskningspersonsformulär)

All data except from the data from the wearables will be collected to an electronic data capture system (eCRF; Smart-Trial) using custom electronic case report forms. A secure link that will lead to the questionnaires will be sent by e-mail to the included patients through Smart-Trial both in pre-specified time points (at baseline and then every three months) but also after each proposed intervention for improving a QoL issues.

It is investigator's responsibility to ensure that data is registered and any corrections in the eCRF are made as stated in the study protocol and in accordance with the instructions and that the registered data is correct, complete, and that reporting takes place according to the timelines that have been predefined and agreed.

Regarding the data from wearables, they will be transferred to the AI-based system using the patient's study code as identification to be merged with the data collected through eCRFs.

12. Notification of study completion, reporting, and publication

Study results will be presented at a group-level using suitable statistical methods. The results will be presented in relevant scientific conferences and published in relevant scientific journals.

The participants will be able to contact the principal investigator if they would like to receive the published results of the study. This information along with the contact information of the primary investigator is included in the informed consent.

13. References

1. Miller KD, Nogueira L, Mariotto AB, et al. Cancer treatment and survivorship statistics, 2019. CA Cancer J Clin. 2019;69:363-385.
2. Nardin S, Mora E, Varughese FM, et al. Breast Cancer Survivorship, Quality of Life, and Late Toxicities. Front Oncol. 2020;10:864.
3. Sanda MG, Dunn RL, Michalski J, et al. Quality of life and satisfaction with outcome among prostate-cancer survivors. N Engl J Med. 2008;358:1250-61.
4. Shakeel S, Tung J, Rahal R, Finley C. Evaluation of Factors Associated With Unmet Needs in Adult Cancer Survivors in Canada. JAMA Netw Open. 2020;3:e200506.
5. Brédart A, Kop JL, Griesser AC, et al. Assessment of needs, health-related quality of life, and satisfaction with care in breast cancer patients to better target supportive care. Ann Oncol. 2013;24:2151-8.
6. Paterson C, Robertson A, Smith A, Nabi G. Identifying the unmet supportive care needs of men living with and beyond prostate cancer: A systematic review. Eur J Oncol Nurs. 2015;19:405-18.
7. Watson E, Shinkins B, Frith E, et al. Symptoms, unmet needs, psychological well-being and health status in survivors of prostate cancer: implications for redesigning follow-up. BJU Int. 2016;117:E10-9.
8. BREAST-Q, “Qportfolio,” 2006. [Online]. Available at: http://qportfolio.org/breast-q/. (Accessed: February 1, 2021)
9. EORTC Quality of Life [Online]. Available at: https://qol.eortc.org/questionnaire/eortc-qlq-c30/ (Accessed: February 1, 2021)
10. EORTC Quality of Life [Online]. Available: https://qol.eortc.org/questionnaire/update-qlq-br23/ (Accessed: February 1, 2021)
11. ePROVIDE [Online]. Available at: https://eprovide.mapi-trust.org/instruments/hospital-anxiety-and-depression-scale (Accessed: Februari 1, 2021)
12. The DASH Outcome Measure [Online]. Available at: https://dash.iwh.on.ca/about-dash (Accessed: February 1, 2021)
13. EORTC Quality of Life [Online]. Available at: https://qol.eortc.org/questionnaire/qlq-pr25/ (Accessed: February 1, 2021)
14. Rosen R, Riley A, Wagner G, et al. The International Index of Erectile Function (IIEF): A multidimensional scale for assessment of erectile dysfunction. Urology 1997;49:822-830.
15. Harrington S, Miale S, Edaugh D. Breast Cancer EDGE Task Force Outcomes: Clinical Measures of Health Related Quality of Life. Rehabilitation Oncology. 2015;33:5–17.
16. Perdomo M, Davies C, Levenhagen K, Ryans K. BREAST CANCER EDGE TASK FORCE OUTCOMES: Assessment Measures of Secondary Lymphedema in Breast Cancer Survivors. Rehabilitation Oncology. 2014;32:22–35.

17. Alappattu M, Harrington SE, Hill A, Roscow A, Jeffrey A. Oncology Section EDGE Task Force on Cancer: A systematic review of patient-reported measures for sexual dysfunction. Rehabil Oncol. 2017;35:137-143.
18. Harrington S, Lee J, Colon G, Alappattu M. Oncology Section EDGE Task Force on Prostate Cancer: A Systematic Review of Outcome Measures for Health-Related Quality of Life. Rehabil Oncol. 2016;34:27-35.

14. Appendix

Appendix 1a. Baseline questionnaire about lifestyle (breast cancer patients)

Bakgrundsfrågor

1. **Vilken är ditt civilstånd?**

☐ Ogift

☐ Gift, registrerat partnerskap eller sammanboende

☐ Skild eller separerat

☐ Änka / änkling

☐ Annat_____________________

2. **Vilken är din högsta avslutade utbildning?**

☐ Grundskola eller motsvarande

☐ Gymnasieexamen eller motsvarande

☐ Eftergymnasial utbildning, ej högskola/universitet

☐ Universitet/högskola

☐ Annat, ange typ av utbildning

3. **Aktuell arbetsform**

☐ Anställd eller egen företagare (heltidsarbete)

☐ Anställd (deltidsarbete)

☐ Arbetssökande

☐ Pensionär

☐ Studerande

4. **Ungefär hur stor är hushållets inkomst före skatt en vanlig månad (SEK)?**

____

Gynekologisk anamnes

5. När fick du din första menstruation?

år

6. När slutade du menstruera?

7. Hur många graviditeter har du haft?

8. Hur många barn har du?

9. Har du genomgått provrörsbefruktning (IVF) tidigare?

☐ Ja

☐ Nej

10. Har du ätit p-piller?

☐ Ja, ange hur många år ☐ Nej

11. Vilket preventivmedel använder du för närvarande?

☐ Preventivmedel utan hormoner (barriärmetoder)

☐ Preventivmedel med hormoner

☐ Hormonspiral

☐ Kopparspiral

☐ Ingen/ej aktuell

12. Har du använt östrogener i samband med klimakteriet?

☐ Ja, ange hur många år ☐ Nej

Frågor om din livstil

13. Röker du?

☐ Ja, ange hur många cigaretter per dag ☐ Nej

14. Är du före detta rökare?

☐ Ja, ange när du slutade röka år ☐ Nej

15. Hur ofta dricker du alkohol?

☐ Aldrig

☐ 1 gång i månaden eller mer sällan

☐ 2-4 gånger i månaden

☐ 2-3 gånger i veckan

☐ 4 gånger/vecka eller mer

Appendix 1b. Baseline questionnaire about lifestyle (prostate cancer patients)

Bakgrundsfrågor

1. **Vilken är ditt civilstånd?**

☐ Ogift

☐ Gift, registrerat partnerskap eller sammanboende

☐ Skild eller separerat

☐ Änka / änkling

☐ Annat______________________

2. **Vilken är din högsta avslutade utbildning?**

☐ Grundskola eller motsvarande

☐ Gymnasieexamen eller motsvarande

☐ Eftergymnasial utbildning, ej högskola/universitet

☐ Universitet/högskola

☐ Annat, ange typ av utbildning

3. **Aktuell arbetsform**

☐ Anställd eller egen företagare (heltidsarbete)

☐ Anställd (deltidsarbete)

☐ Arbetssökande

☐ Pensionär

☐ Studerande

4. **Ungefär hur stor är hushållets inkomst före skatt en vanlig månad (SEK)?**

____

Frågor om din livstil

5. Röker du?

☐ Ja, ange hur många cigaretter per dag ☐ Nej

6. Är du före detta rökare?

☐ Ja, ange när du slutade röka år ☐ Nej

7. Hur ofta dricker du alkohol?

☐ Aldrig

☐ 1 gång i månaden eller mer sällan

☐ 2-4 gånger i månaden

☐ 2-3 gånger i veckan

☐ 4 gånger/vecka eller mer

Appendix 2. Questionnaire about nutrition

Frågor om dina matvanor

Tänk på vad du har ätit och druckit **de senaste veckorna** och besvara följande frågor genom att ringa numret som passar bäst

1. **Hur skulle du generellt skatta dina matvanor kring att äta hälsosamt?**

Dåligt	Mindre bra	Bra	Mycket bra	Utomordentligt
1	2	3	4	5

2. **Hur många gånger per dag har du ätit snabb mat eller snacks med höga halter av fett, socker, eller salt de senaste veckorna?**

Minst 6 gånger	4 - 5 gånger	2 - 3 gånger	1 gång	Mindre än en gång
1	2	3	4	5

3. **Hur många portioner (en portion = en halv kopp) frukt per dag har du ätit de senaste veckorna?**

Mindre än en	1 portion	2 - 3 portioner	4 - 5 portioner	Minst 6 portioner
1	2	3	4	5

4. **Hur många portioner (en portion = en halv kopp) grönsaker per dag har du ätit de senaste veckorna?**

Mindre än en	1 portion	2 - 3 portioner	4 - 5 portioner	Minst 6 portioner
1	2	3	4	5

5. **Hur många gånger per dag har du druckit läsk (inklusive sötat kaffe, energidryck, icke-färskpressad juice) de senaste veckorna?**

Minst 6 gånger	4 - 5 gånger	2 - 3 gånger	1 gång	Mindre än en gång
1	2	3	4	5

6. **Hur många gånger per dag har du ätit chips eller kex de senaste veckorna?**

Minst 6 gånger	4 - 5 gånger	2 - 3 gånger	1 gång	Mindre än en gång
1	2	3	4	5

7. **Hur många gånger per dag har du ätit godis eller sötsaker (inklusive glass) de senaste veckorna?**

Minst 6 gånger	4 - 5 gånger	2 - 3 gånger	1 gång	Mindre än en gång
1	2	3	4	5

8. Hur mycket margarin, smör eller matolja använder du i din mat de senaste veckorna?

Höga mängder	Mycket	En hel del	Lite	Inte alls
1	2	3	4	5

9. Hur många gånger per dag har du ätit mejeriprodukter de senaste veckorna?

Mindre än en gång	1 gång	2 - 3 gånger	4 - 5 gånger	Minst 6 gånger
1	2	3	4	5

10. Hur många gånger per dag har du ätit kött eller fisk de senaste veckorna?

Mindre än en gång	1 gång	2 - 3 gånger	4 - 5 gånger	Minst 6 gånger
1	2	3	4	5

Appendix 3a. Breast-Q mastectomy

BREAST-Q™ – BREAST CANCER CORE SCALE (PRE- AND POSTOPERATIVE) VERSION 2.0: SATISFACTION WITH BREASTS

Med tanke på ditt bröstområde, hur nöjd eller missnöjd har du under den senaste veckan varit med:

	Mycket missnöjd	Ganska missnöjd	Ganska nöjd	Mycket nöjd
a. Hur du ser ut i spegeln påklädd?	1	2	3	4
b. Hur bekvämt dina bh:ar sitter?	1	2	3	4
c. Möjligheten att kunna bära mer figurnära kläder?	1	2	3	4
d. Hur du ser ut i spegeln naken?	1	2	3	4

BREAST-Q VERSION 2.0 © Memorial Sloan Kettering Cancer Center and The University of British Columbia, 2017. All rights reserved.

BREAST-Q™ – BREAST CANCER CORE SCALE (PRE- AND POSTOPERATIVE) VERSION 2.0: PSYCHOSOCIAL WELL-BEING

Med tanke på ditt bröstområde, hur ofta har du under de senaste den senaste veckan:

	Aldrig	Några få ganger	Ganska ofta	Nästan jämt	Hela tiden
a. Känt självförtroende i sociala sammanhang?	1	2	3	4	5
b. Varit känslomässigt kapabel att göra det du vill?	1	2	3	4	5
c. Varit känslomässigt stabil?	1	2	3	4	5
d. Känt att du har samma värde som andra kvinnor?	1	2	3	4	5
e. Känt dig självsäker?	1	2	3	4	5
f. Känt dig feminin, påklädd?	1	2	3	4	5
g. Känt att du accepterar din kropp?	1	2	3	4	5
h. Känt dig normal?	1	2	3	4	5
i. Känt dig som andra kvinnor?	1	2	3	4	5
j. Känt dig attraktiv?	1	2	3	4	5

BREAST-Q VERSION 2.0 © Memorial Sloan Kettering Cancer Center and The University of British Columbia, 2017. All rights reserved.

BREAST-Q™ – BREAST CANCER CORE SCALE (PRE- AND POSTOPERATIVE) VERSION 2.0: SEXUAL WELL-BEING

Med tanke på din sexualitet, hur ofta känner du dig i allmänhet:

	Aldrig	Några få gånger	Ganska ofta	Nästan jämt	Hela tiden
a. Sexuellt attraktiv påklädd?	1	2	3	4	5
b. Bekväm/avslappnad i samband med sexuell aktivitet?	1	2	3	4	5
c. Sexuellt trygg?	1	2	3	4	5
d. Nöjd med ditt sexliv?	1	2	3	4	5
e. Sexuellt trygg med hur dina bröst ser ut utan kläder?	1	2	3	4	5
f. Sexuellt attraktiv naken?	1	2	3	4	5

BREAST-Q VERSION 2.0 © Memorial Sloan Kettering Cancer Center and The University of British Columbia, 2017. All rights reserved.

BREAST-Q™ – BREAST CANCER CORE SCALE (PRE- AND POSTOPERATIVE) VERSION 2.0: PHYSICAL WELL-BEING: CHEST

Hur ofta har du under den senaste veckan?

	Aldrig	Ganska ofta	Hela tiden
a. Känt smärta i bröstmusklerna?	**1**	**2**	**3**
b. Haft svårt att lyfta eller röra dina armar?	**1**	**2**	**3**
c. Haft svårt att sova på grund av obehag i bröstområdet?	**1**	**2**	**3**
d. Känt att det spänner/stramar over bröstområdet?	**1**	**2**	**3**
e. Känt något som drar i bröstområdet?	**1**	**2**	**3**
f. Haft en irriterande känsla i bröstområdet?	**1**	**2**	**3**
g. Känt ömhet i bröstområdet?	**1**	**2**	**3**
h. Haft skarpa smärtor i bröstområdet?	**1**	**2**	**3**
i. Haft värk i bröstområdet?	**1**	**2**	**3**
j. Haft en pulserande känsla i bröstområdet?	**1**	**2**	**3**

Post-operative only

k. Haft svullnad (lymfödem) av armen på den sida som du opererat ditt bröst?	**1**	**2**	**3**

BREAST-Q VERSION 2.0 © Memorial Sloan Kettering Cancer Center and The University of British Columbia, 2017. All rights reserved.

Appendix 3b. Breast-Q breast conserving therapy

BREAST-Q™ – BREAST CANCER CORE SCALE (PRE- AND POSTOPERATIVE) VERSION 2.0: PSYCHOSOCIAL WELL-BEING

Med tanke på dina bröst, hur ofta har du under den senaste veckan:

	Aldrig	Några få gånger	Ganska ofta	Nästan jämt	Hela tiden
a. Känt självförtroende i sociala sammanhang?	1	2	3	4	5
b. Varit känslomässigt kapabel att göra det du vill?	1	2	3	4	5
c. Varit känslomässigt stabil?	1	2	3	4	5
d. Känt att du har samma värde som andra kvinnor	1	2	3	4	5
e. Känt dig självsäker?	1	2	3	4	5
f. Känt dig feminin, påklädd	1	2	3	4	5
g. Känt att du accepterar din kropp?	1	2	3	4	5
h. Känt dig normal?	1	2	3	4	5
i. Känt dig som andra kvinnor?	1	2	3	4	5
j. Känt dig attraktiv?	1	2	3	4	5

BREAST-Q VERSION 2.0 © Memorial Sloan Kettering Cancer Center and The University of British Columbia, 2017. All rights reserved.

BREAST-Q™ - BREAST CANCER CORE SCALE (PRE- AND POSTOPERATIVE) VERSION 2.0: SEXUAL WELL-BEING

Med tanke på din sexualitet, hur ofta känner du dig i allmänhet:

	Aldrig	Några få gånger	Ganska ofta	Nästan jämt	Hela tiden
a. Sexuellt attraktiv påklädd?	1	2	3	4	5
b. Bekväm/avslappnad i samband med sexuell aktivitet?	1	2	3	4	5
c. Sexuellt trygg?	1	2	3	4	5
d. Nöjd med ditt sexliv?	1	2	3	4	5
e. Sexuellt trygg med hur dina bröst ser ut utan kläder?	1	2	3	4	5
f. Sexuellt attraktiv naken?	1	2	3	4	5

BREAST-Q VERSION 2.0 © Memorial Sloan Kettering Cancer Center and The University of British Columbia, 2017. All rights reserved.

BREAST-Q™ - BREAST CANCER CORE SCALE (PREOPERATIVE) VERSION 2.0: SATISFACTION WITH BREASTS

Med tanke på dina bröst, hur nöjd eller missnöjd har du under den senaste veckan varit med:

	Mycket missnöjd	Ganska missnöjd	Ganska nöjd	Mycket nöjd
a. Hur du ser ut i spegeln påklädd?	1	2	3	4
b. Hur bekvämt dina bh:ar sitter?	1	2	3	4
c. Möjligheten att kunna bära mer figurnära kläder?	1	2	3	4
d. Hur du ser ut i spegeln naken?	1	2	3	4

BREAST-Q VERSION 2.0 © Memorial Sloan Kettering Cancer Center and The University of British Columbia, 2017. All rights reserved.

BREAST-Q™ – BCT MODULE (POSTOPERATIVE) VERSION 2.0: SATISFACTION WITH BREASTS

Följande frågor handlar om dina bröst och din behandling för bröstcancer (med behandling menas operation med bröstbevarande kirurgi, med eller utan strålbehandling). Om du har behandlats med bröstbevarande kirurgi och strålningsbehandling på båda brösten, svara på frågorna med tanke på det bröst du är minst nöjd med. Med tanke på dina bröst, hur nöjd eller missnöjd har du under den senaste veckan varit med:

	Mycket missnöjd	Ganska missnöjd	Ganska nöjd	Mycket nöjd
a. Hur du ser ut i spegeln påklädd?	1	2	3	4
b. Formen på ditt opererade bröst när du bär bh?	1	2	3	4
c. Hur normal du känner dig med kläderna på?	1	2	3	4
d. Möjligheten att kunna bära mer figurnära kläder?	1	2	3	4
e. Hur naturligt ditt opererade bröst sitter/hänger på bröstkorgen?	1	2	3	4
f. Hur mjukt format ditt opererade bröst är?	1	2	3	4
g. Konturen på ditt opererade bröst?	1	2	3	4
h. Hur jämnstora dina bröst är?	1	2	3	4
i. Hur naturligt ditt opererade bröst ser ut?	1	2	3	4
j. Hur pass lika dina bröst är varandra?	1	2	3	4
k. Hur du ser ut i spegeln naken?	1	2	3	4

BREAST-Q® VERSION 2.0 © Memorial Sloan Kettering Cancer Center and The University of British Columbia, 2017. All rights reserved.

BREAST-Q™ - BREAST CANCER CORE SCALE (PREOPERATIVE) VERSION 2.0: PHYSICAL WELL-BEING: CHEST

Hur ofta har du under de senaste den senaste veckan :

	Aldrig	Ganska ofta	Hela tiden
a. Känt muskelsmärta i bröstkorgen?	1	2	3
b. Haft svårt att lyfta eller röra dina armar?	1	2	3
c. Haft svårt att sova på grund av obehag i bröstområdet?	1	2	3
d. Känt att det spänner/stramar över bröstområdet?	1	2	3
e. Känt något som drar i bröstområdet?	1	2	3
f. Känt molande smärta i bröstområdet?	1	2	3
g. Känt ömhet i bröstområdet?	1	2	3
h. Haft skarpa smärtor i bröstområdet?	1	2	3
i. Haft värk i bröstområdet?	1	2	3
j. Upplevt att det bultar i bröstområdet?	1	2	3

BREAST-Q VERSION 2.0 © Memorial Sloan Kettering Cancer Center and The University of British Columbia, 2017. All rights reserved.

**BREAST-Q™ - BCT MODULE (POSTOPERATIVE) VERSION 2.0:
PHYSICAL WELL-BEING: CHEST**

Hur ofta har du under den senaste veckan:

	Aldrig	Några få gånger	Hela tiden
a. Haft svårt att lyfta eller röra på dina armar?	1	2	3
b. Haft svårt att sova på grund av obehag från ditt opererade bröst?	1	2	3
c. Känt att det spänner/stramar i brösmområdet?	1	2	3
d. Känt något som drar i bröstområdet?	1	2	3
e. Känt ömhet i bröstområdet?	1	2	3
f. Haft skarpa smärtor i bröstområdet?	1	2	3
g. Känt värk i bröstområdet?	1	2	3
h. Haft svårt att ligga på den sida som du opererat ditt bröst?	1	2	3
i. Haft svullnad (lymfödem) av armen på den sida som du opererat ditt bröst?	1	2	3

BREAST-Q® VERSION 2.0 © Memorial Sloan Kettering Cancer Center and The University of British Columbia, 2017. All rights reserved.

**BREAST-Q™ – BREAST CANCER CORE SCALE (POSTOPERATIVE) VERSION 2.0:
ADVERSE EFFECTS OF RADIATION**

Om du fick strålningsbehandling på båda brösten, svara på frågorna med tanke på det bröst du är minst nöjd med. Med tanke på ditt/dina strålade bröst, hur mycket har du under den senaste veckan bekymrats av:

	Inte alls	Lite	Mycket
a. Att huden på ditt strålade bröst ser annorlunda ut (t ex alltför mörk eller alltför ljus)?	1	2	3
b. Märken på huden på ditt bröst orsakade av strålning (t ex små synliga blodkärl)?	1	2	3
c. Att huden på ditt strålade bröst känns torr?	1	2	3
d. Att huden på ditt strålade bröst känns öm (känslig) när den berörs (t ex av förändrad vattentemperatur då du duschar/badar)?	1	2	3
e. Att huden på ditt strålade bröst känns onaturligt tjock (grov,hård) när du rör den?	1	2	3
f. Att huden på ditt strålade bröst känns irriterad av dina kläder?	1	2	3

BREAST-Q® VERSION 2.0 © Memorial Sloan Kettering Cancer Center and The University of British Columbia, 2017. All rights reserved.

Appendix 4. EORTC-QLQ30 questionnaire

SWEDISH

EORTC QLQ-C30 (version 3)

Vi är intresserade av några saker som har med dig och din hälsa att göra. Besvara alla frågor genom att sätta en ring runt den siffra som stämmer bäst in på dig. Det finns inga svar som är "rätt" eller "fel". Den information du lämnar kommer att hållas strikt konfidentiell.

Fyll i dina initialer: ⌊_⌊_⌊_⌊_⌋
När är du född (Dag, Månad, År): ⌊_⌊_⌊_⌊_⌊_⌊_⌊_⌊_⌋
Dagens datum (Dag, Månad, År): 31 ⌊_⌊_⌊_⌊_⌊_⌊_⌊_⌊_⌋

		Inte alls	Lite	En hel del	Mycket
1.	Har du svårt att göra ansträngande saker, som att bära en tung kasse eller väska?	1	2	3	4
2.	Har du svårt att ta en <u>lång</u> promenad?	1	2	3	4
3.	Har du svårt att ta en <u>kort</u> promenad utomhus?	1	2	3	4
4.	Måste du sitta eller ligga på dagarna?	1	2	3	4
5.	Behöver du hjälp med att äta, klä dig, tvätta dig eller gå på toaletten?	1	2	3	4

Under veckan som gått:

		Inte alls	Lite	En hel del	Mycket
6.	Har du varit begränsad i dina möjligheter att utföra antingen ditt förvärvsarbete eller andra dagliga aktiviteter?	1	2	3	4
7.	Har du varit begränsad i dina möjligheter att utöva dina hobbyer eller andra fritidssysselsättningar?	1	2	3	4
8.	Har du blivit andfådd?	1	2	3	4
9.	Har du haft ont?	1	2	3	4
10.	Har du behövt vila?	1	2	3	4
11.	Har du haft svårt att sova?	1	2	3	4
12.	Har du känt dig svag?	1	2	3	4
13.	Har du haft dålig aptit?	1	2	3	4
14.	Har du känt dig illamående?	1	2	3	4
15.	Har du kräkts?	1	2	3	4
16.	Har du varit förstoppad?	1	2	3	4

<u>Fortsätt på nästa sida</u>

SWEDISH

**Under veckan som gått:**	**Inte alls**	**Lite**	**En hel del**	**Mycket**
17. Har du haft diarré?	1	2	3	4
18. Har du varit trött?	1	2	3	4
19. Har dina dagliga aktiviteter påverkats av smärta?	1	2	3	4
20. Har du haft svårt att koncentrera dig på saker som att läsa en tidning eller titta på TV?	1	2	3	4
21. Har du känt dig spänd?	1	2	3	4
22. Har du oroat dig?	1	2	3	4
23. Har du känt dig irriterad?	1	2	3	4
24. Har du känt dig nedstämd?	1	2	3	4
25. Har du haft svårt att komma ihåg saker?	1	2	3	4
26. Har ditt fysiska tillstånd eller den medicinska behandlingen stört ditt familjeliv?	1	2	3	4
27. Har ditt fysiska tillstånd eller den medicinska behandlingen stört dina sociala aktiviteter?	1	2	3	4
28. Har ditt fysiska tillstånd eller den medicinska behandlingen gjort att du fått ekonomiska svårigheter?	1	2	3	4

Sätt en ring runt den siffra mellan 1 och 7 som stämmer bäst in på dig för följande frågor:

29. Hur skulle du vilja beskriva din hälsa totalt sett under den vecka som gått?

1	2	3	4	5	6	7
Mycket dålig						Utmärkt

30. Hur skulle du vilja beskriva din totala livskvalitet under den vecka som gått?

1	2	3	4	5	6	7
Mycket dålig						Utmärkt

© QLQ-C30 Copyright 1995 EORTC Quality of Life Group. Alla rättigheter reserverade. Version 3.0.

Appendix 5. EORTC-BR23 questionnaire

SWEDISH

EORTC QLQ - BR23

Patienter berättar ibland att de har följande symptom eller problem. Markera i vilken utsträckning som du har haft dessa symptom eller problem under den senaste veckan. Svara genom att ringa in den siffra som bäst passar in på dig.

Under veckan som gått:	Inte alls	Lite	En hel del	Mycket
31. Har du varit torr i munnen?	1	2	3	4
32. Har mat och dryck smakat annorlunda än vanligt?	1	2	3	4
33. Har ögonen varit irriterade, runnit eller gjort ont?	1	2	3	4
34. Har du tappat hår?	1	2	3	4
35. Besvara denna fråga endast om du tappat hår: Blev du illa berörd över hårförlusten?	1	2	3	4
36. Har du känt dig sjuk eller dålig?	1	2	3	4
37. Har du haft svettningar och blodvallningar?	1	2	3	4
38. Har du haft huvudvärk?	1	2	3	4
39. Har du känt dig mindre attraktiv på grund av sjukdomen eller behandlingen?	1	2	3	4
40. Har du känt dig mindre kvinnlig på grund av sjukdomen eller behandlingen?	1	2	3	4
41. Har du känt det svårt att se dig själv naken?	1	2	3	4
42. Har du känt dig missnöjd med din kropp?	1	2	3	4
43. Har du oroat dig för din hälsa i framtiden?	1	2	3	4

Under de senaste fyra veckorna:	Inte alls	Lite	En hel del	Mycket
44. I vilken utsträckning har du varit intresserad av sex?	1	2	3	4
45. I vilken utsträckning har du varit sexuellt aktiv? (med eller utan samlag)	1	2	3	4
46. Besvara denna fråga endast om du varit sexuellt aktiv: I vilken utsträckning har sex varit till glädje för dig?	1	2	3	4

Fortsätt på nästa sida

SWEDISH

Under veckan som gått:	Inte alls	Lite	En hel del	Mycket
47. Har du haft ont i armen eller axeln?	1	2	3	4
48. Har din arm eller hand varit svullen?	1	2	3	4
49. Har det varit svårt att lyfta armen eller röra den i sidled?	1	2	3	4
50. Har du haft ont i eller omkring det sjuka bröstet?	1	2	3	4
51. Har du varit svullen i eller omkring det sjuka bröstet?	1	2	3	4
52. Har området kring det sjuka bröstet varit extra känsligt?	1	2	3	4
53. Har du haft hudbesvär i eller omkring det sjuka bröstet (t.ex. om det har kliat, varit torrt eller fjällat)?	1	2	3	4

© QLQ-BR23 Copyright 1994 EORTC Quality of Life Group. Alla rättigheter reserverade. Version 1.0

Appendix 6. HADS questionnaire

Hospital Anxiety and Depression Scale (HAD)

Frågor som relaterar till ångest är markerade med Å och depression med D. Poängen för varje svar finns i högerkolumnen. Markera svaren på respektive fråga och summera på slutet av sidan.
Ringa in de svar som du tycker passar bäst.

Å Jag känner mig spänd och nervös:

Mestadels	3
Ofta	2
Av och till	1
Inte alls	0

D Jag uppskattar fortfarande saker jag tidigare uppskattat:

Definitivt lika mycket	0
Inte lika mycket	1
Endast delvis	2
Nästa inte alls	3

Å Jag har en känsla av att något hemskt kommer att hända:

Mycket klart och obehagligt	3
Inte så starkt nu	2
Betydligt svagare nu	1
Inte alls	0

D Jag kan skratta och se det roliga i saker och ting:

Lika ofta som tidigare	0
Inte lika ofta nu	1
Betydligt mer sällan nu	2
Aldrig	3

Å Jag bekymrar mig över saker:

Mestadels	3
Ganska ofta	2
Av och till	1
Någon enstaka gång	0

D Jag känner mig på gott humör:

Aldrig	3
Sällan	2
Ibland	1
Mestadels	0

Å Jag kan sitta stilla och känna mig avslappnad:

Absolut	0
Vanligtvis	1
Sällan	2
Aldrig	3

D Allting känns trögt:

Nästan alltid	3
Ofta	2
Ibland	1
Aldrig	0

Å Jag känner mig orolig, som om jag hade fjärilar i magen:

Aldrig	0
Ibland	1
Ganska ofta	2
Väldigt ofta	3

D Jag har tappat intresset för hur jag ser ut:

Fullständigt	3
Till stor del	2
Delvis	1
Inte alls	0

Å Jag känner mig rastlös:

Väldigt ofta	3
Ganska ofta	2
Sällan	1
Inte alls	0

D Jag ser med glädje fram emot saker och ting:

Lika mycket som tidigare	0
Mindre än tidigare	1
Mycket mindre än tidigare	2
Knappast alls	3

Å Jag får plötsliga panikkänslor:

Väldigt ofta	3
Ganska ofta	2
Sällan	1
Aldrig	0

D Jag kan uppskatta en god bok, ett TV- eller radioprogram:

Ofta	0
Ibland	1
Sällan	2
Mycket sällan	3

Totalt Å =______
Totalt D =______

Appendix 7. DASH questionnaire

Hälsoenkät (arm/axel/hand)

sida 1/3

Denna enkät berör Dina symtom och Din förmåga att utföra vissa aktiviteter.
Svara på **varje fråga**, baserat på hur Du har mått **den senaste veckan**, genom att kryssa för ett svarsalternativ för varje fråga.
Om det är någon aktivitet Du inte har utfört den senaste veckan får Du kryssa för det svar som Du bedömer **stämmer bäst** om Du hade utfört aktiviteten.
Det har ingen betydelse vilken arm eller hand Du använder för att utföra aktiviteten. Svara baserat på Din förmåga oavsett hur Du utför uppgiften.

	Ingen svårighet	Viss svårighet	Måttlig svårighet	Stor svårighet	Omöjligt att göra
1. Öppna en ny burk, eller hårt sittande lock	☐	☐	☐	☐	☐
2. Skriva	☐	☐	☐	☐	☐
3. Vrida om en nyckel	☐	☐	☐	☐	☐
4. Förbereda en måltid	☐	☐	☐	☐	☐
5. Öppna en tung dörr	☐	☐	☐	☐	☐
6. Lägga upp något på en hylla över Ditt huvud	☐	☐	☐	☐	☐
7. Utföra tunga hushållssysslor (t ex tvätta golv och väggar, putsa fönster, hänga tvätt)	☐	☐	☐	☐	☐
8. Trädgårdsarbete	☐	☐	☐	☐	☐
9. Bädda sängen	☐	☐	☐	☐	☐
10. Bära matkassar eller portfölj	☐	☐	☐	☐	☐
11. Bära tunga saker (över fem kilo)	☐	☐	☐	☐	☐
12. Byta en glödlampa ovanför Ditt huvud	☐	☐	☐	☐	☐
13. Tvätta eller föna håret	☐	☐	☐	☐	☐
14. Tvätta Din rygg	☐	☐	☐	☐	☐
15. Ta på en tröja	☐	☐	☐	☐	☐
16. Använda en kniv för att skära upp maten	☐	☐	☐	☐	☐
17. Fritidsaktiviteter som kräver liten ansträngning (t ex spela kort, sticka, boule)	☐	☐	☐	☐	☐
18. Fritidsaktiviteter som tar upp viss kraft eller stöt genom arm, axel eller hand (t ex spela golf, använda hammare, spela tennis, skytte, bowling)	☐	☐	☐	☐	☐
19. Friritidsaktiviteter där Du rör på armen fritt (t ex spela badminton, simma, gympa)	☐	☐	☐	☐	☐
20. Färdas från en plats till en annan	☐	☐	☐	☐	☐
21. Sexuella aktiviteter	☐	☐	☐	☐	☐

22. Under **de senaste sju dagarna**, i vilken utsträckning har Dina arm-, axel- eller handproblem stört Ditt vanliga umgänge med anhöriga, vänner, grannar eller andra?

☐ Inte alls ☐ Lite ☐ Måttligt ☐ Mycket ☐ Väldigt mycket

23. Under **de senaste sju dagarna**, i vilken utsträckning har Dina arm-, axel- eller handproblem stört Ditt vanliga arbete eller andra dagliga aktiviteter?

☐ Inte alls ☐ Lite ☐ Måttligt ☐ Mycket ☐ Väldigt mycket

Ange svårighetsgraden på Dina symtom **de senaste sju dagarna**:

	Ingen	Lätt	Måttlig	Svår	Mycket svår
24. Värk/smärta i arm, axel eller hand	☐	☐	☐	☐	☐
25. Värk/smärta i arm, axel eller hand i samband med aktivitet	☐	☐	☐	☐	☐
26. Stickningar (sockerdrickskänsla) i arm, axel eller hand	☐	☐	☐	☐	☐
27. Svaghet i arm, axel eller hand	☐	☐	☐	☐	☐
28. Stelhet i arm, axel eller hand	☐	☐	☐	☐	☐

29. Har Du haft svårt att sova, under **de senaste sju dagarna**, på grund av värk/smärta i arm, axel eller hand?

☐ Inte alls ☐ Viss svårighet ☐ Måttlig svårighet ☐ Stor svårighet ☐ Mycket stor svårighet

30. Jag känner mig mindre kapabel, har sämre självförtroende eller känner mig mindre behövd på grund av mina arm-, axel- eller handproblem.

☐ Instämmer absolut inte ☐ Instämmer inte ☐ Vet inte ☐ Instämmer ☐ Instämmer absolut

Följande frågor rör hur mycket Dina arm-, axel- eller handproblem påverkat Din förmåga att arbeta (inklusive hushållsarbete om detta är Ditt huvudsakliga arbete).

Arbetar Du? ☐ Ja ☐ Nej

Om Du inte arbetar kan Du hoppa över de följande fyra frågorna

Ange här Ditt arbete ______

Kryssa för det påstående som bäst stämmer in på Din kroppsliga förmåga **de senaste sju dagarna**.

Hade Du någon svårighet att:	Ingen svårighet	Viss svårighet	Måttlig svårighet	Stor svårighet	Omöjligt
1. använda Din vanliga teknik för att arbeta?	☐	☐	☐	☐	☐
2. utföra Ditt ordinarie arbete på grund av värk/smärta i arm, axel eller hand?	☐	☐	☐	☐	☐
3. utföra Ditt arbete så bra som Du skulle vilja?	☐	☐	☐	☐	☐
4. utföra Ditt arbete på den tid Du brukar använda?	☐	☐	☐	☐	☐

Följande frågor rör hur mycket Dina arm-, axel- eller handproblem påverkat Din förmåga att spela musikinstrument och/eller utöva idrott.

Spelar Du något musikinstrument eller utövar någon idrott? ☐ Ja ☐ Nej

Om Du inte spelar något musikinstrument eller utövar någon idrott kan Du hoppa över resterande frågor

Om Du spelar mer än ett musikinstrument eller utövar mer än en idrott ska Du svara med avseende på den aktivitet som är viktigast för Dig.
Ange här det musikinstrument eller den idrott som är viktigast för Dig ______

Kryssa för det påstående som bäst stämmer in på Din kroppsliga förmåga **de senaste sju dagarna**.

Hade Du någon svårighet att:	Ingen svårighet	Viss svårighet	Måttlig svårighet	Stor svårighet	Omöjligt
1. använda Din vanliga teknik för att spela instrument/idrotta?	☐	☐	☐	☐	☐
2. spela instrument/idrotta på grund av värk/smärta i arm, axel eller hand?	☐	☐	☐	☐	☐
3. spela instrument/idrotta så bra som Du skulle vilja?	☐	☐	☐	☐	☐
4. använda lika mycket tid som vanligt för att spela instrument/idrotta?	☐	☐	☐	☐	☐

Appendix 8. EORTC PR25 questionnaire

SWEDISH

EORTC QLQ - PR25

Patienter berättar ibland att de har följande symptom. Markera i vilken utsträckning som du har haft dessa symptom under den senaste veckan. Svara genom att ringa in den siffra som bäst passar in på dig.

Under veckan som gått:	Inte alls	Lite	En hel del	Mycket
31. Har du behövt urinera ofta **under dagen**?	1	2	3	4
32. Har du behövt urinera ofta **under natten?**	1	2	3	4
33. Måste du skynda dig till toaletten när du kände att du behövde urinera?	1	2	3	4
34. Har du haft svårt att få tillräckligt med sömn för att du måste stiga upp ofta om natten och urinera?	1	2	3	4
35. Har det varit besvärligt att gå hemifrån, för att du måste vara i närheten av en toalett hela tiden?	1	2	3	4
36. Har du haft urinläckage?	1	2	3	4
37. Har du känt smärta eller sveda i samband med urinering?	1	2	3	4
38. Besvara denna fråga endast om du använder ett inkontinensskydd: Har det inneburit ett problem för dig att använda inkontinensskydd?	1	2	3	4
39. Har dina dagliga rutiner begränsats av urinvägsproblem?	1	2	3	4
40. Har dina dagliga rutiner begränsats av tarmproblem?	1	2	3	4
41. Har du haft oavsiktlig avföring (läckage)?	1	2	3	4
42. Har du haft blod i avföringen?	1	2	3	4
43. Har du känt dig uppsvälld i magen?	1	2	3	4
44. Har du haft svettningar och blodvallningar?	1	2	3	4
45. Har dina bröstvårtor eller bröst blivit större eller känts ömma?	1	2	3	4
46. Har dina ben eller vrister varit svullna?	1	2	3	4

Fortsätt på nästa sida

SWEDISH

**Under de senaste fyra veckorna:**	**Inte alls**	**Lite**	**En hel del**	**Mycket**
47. Har du haft problem med viktminskning?	1	2	3	4
48. Har du haft problem med viktökning?	1	2	3	4
49. Har sjukdomen och/eller din behandling påverkat din manlighet?	1	2	3	4
50. Har du varit intresserad av sex?	1	2	3	4
51. Har du varit sexuellt aktiv (med eller utan samlag)?	1	2	3	4

BESVARA DE FÖLJANDE FYRA FRÅGORNA ENDAST OM DU HAR VARIT SEXUELLT AKTIV UNDER DE SENASTE 4 VECKORNA:

52. Har du kunnat njuta av sex?	1	2	3	4
53. Har du haft svårt att få eller bibehålla en erektion?	1	2	3	4
54. Har du haft problem med utlösning (t.ex. "torr" utlösning, dvs orgasm utan sädesuttömning)?	1	2	3	4
55. Har du känt dig besvärad i sexuellt intima situationer?	1	2	3	4

© Copyright 1999 EORTC Quality of Life Study Group. Alla rättigheter förbehålles.

Appendix 9. International Index of Erectile Function (IIEF) questionnaire

IIEF-5 ”ED-SCORE”

Varje fråga har *5 svarsalternativ.* Dessutom finns i de flesta fall ytterligare en kolumn med ett kryss (X) som du ringar in om frågan inte är relevant för Dig. Ringa in det svar som bäst beskriver din situation. Ringa endast in *ett* svarsalternativ per fråga.

**EREKTION**		Mycket svag eller ingen alls	Svag	Måttlig	Stark	Mycket stark
1. Hur uppskattar Du att din *tilltro* till att kunna få och behålla en erektion varit de senaste 6 månaderna?		1	2	3	4	5
2. *Hur ofta* efter sexuell stimulering har Din erektion, under de senaste 6 månaderna, blivit *tillräckligt styv* för att kunna tränga in i din partner?	Ingen sexuell aktivitet har förekommit	Nästan aldrig eller aldrig	Några få gånger (mycket färre än hälften av gångerna)	Ibland (ungefär hälften av gångerna)	De flesta gångerna (mycket mer än hälften av gångerna)	Nästan alltid eller alltid
	X	1	2	3	4	5
3. *Hur ofta* har Du, under samlag, *kunnat behålla* erektionen sedan Du trängt in i Din partner de senaste 6 månaderna?	Inga försök till samlag har förekommit	Nästan aldrig eller aldrig	Några få gånger (mycket färre än hälften av gångerna)	Ibland (ungefär hälften av gångerna)	De flesta gångerna (mycket mer än hälften av gångerna)	Nästan alltid eller alltid
	X	1	2	3	4	5
4. *Hur svårt* hade Du att behålla erektionen ända till slutet av samlaget de senaste 6 månaderna?	Inga försök till samlag har förekommit	Mycket stora svårigheter	Stora svårigheter	Svårigheter	Vissa svårigheter	Inga svårigheter
	X	1	2	3	4	5
**TILLFREDS-STÄLLELSE**	Inga försök till samlag har förekommit	Nästan aldrig eller aldrig	Några få gånger (mycket färre än hälften av gångerna)	Ibland (ungefär hälften av gångerna)	De flesta gångerna (mycket mer än hälften av gångerna)	Nästan alltid eller alltid
5. När Du *de senaste 6 månaderna* försökt genomföra samlag, hur ofta har *Du* upplevt dem som tillfredsställande?	X	1	2	3	4	5

Total ED-poäng (fr 1–5): ______________

Appendix 10. List of interventions for specific QoL issues

Breast Cancer: Anxiety

- Mindfulness
- Nordic Walking
- Anti-stress techniques
- Promotion of positive familiar and social relationships
- Psychological support
- Physical exercise
- Anxiolytic medication
- Mirtazapine

Breast Cancer: Depression

- Mindfulness
- Nordic Walking
- Anti-stress techniques
- Promotion of positive familiar and social relationships
- Psychological support
- Physical exercise
- Psychiatric support
- SSRIs
- SNRIs
- Tricyclic antidepressants

Breast Cancer: Fatigue

- Physical activity
- Cognitive behavioural therapy
- Mindfulness-based stress reduction
- Yoga
- Psycho-educational therapies
- Supportive expressive therapy
- Nutrition consultation
- Interventions for sleep disturbances

Breast Cancer: Weight gain

- Diet
- Physical activity
- Integrated diet and physical activity
- Consultation with a nutritionist

Breast Cancer: Hot flushes

- Physical activity
- Movement-based relaxation techniques
- Cognitive behavioural therapy
- Antidepressants (venlafaxine 1st choice; paroxetine and citalopram 2nd choice)
- Neuroleptic agents (gabapentin 1st choice; pregabalin 2nd choice)
- Oxibutynine (if other pharmacological approaches have no effect)

Breast Cancer: Joint Pain

- Physical activity
- Movement-based relaxation techniques
- Acupuncture
- Switch aromatase inhibitors
- Switch from aromatase inhibitors to tamoxifen
- Analgesic (NSAID)
- Duloxetine

Breast Cancer: Neurotoxicity
- Physical activity
- Acupuncture
- Scrambler therapy
- Duloxetine

Prostate Cancer: Anxiety
- Mindfulness
- Nordic Walking
- Anti-stress techniques
- Promotion of positive familiar and social relationships
- Psychological support
- Physical exercise
- Anxiolytic medication
- Mirtazapine

Prostate Cancer: Depression
- Mindfulness
- Nordic Walking
- Anti-stress techniques
- Promotion of positive familiar and social relationships
- Psychological support
- Physical exercise
- Psychiatric support
- SSRIs
- SNRIs
- Tricyclic antidepressants

Prostate Cancer: Fatigue
- Physical activity
- Cognitive behavioural therapy
- Mindfulness-based stress reduction
- Yoga
- Psycho-educational therapies
- Supportive expressive therapy
- Nutrition consultation
- Interventions for sleep disturbances

Prostate Cancer: Weight changes
- Diet
- Physical activity

- Integrated diet and physical activity

Prostate Cancer: Hot flushes

- Physical activity
- Movement-based relaxation techniques
- Cognitive behavioural therapy
- Progesterone/progestin
- Cyproterone
- Antidepressants (venlafaxine 1st choice; paroxetine 2nd choice)
- Neuroleptic agents (gabapentin)
- Oxibutynine (if other pharmacological approaches have no effect)
- Androgen deprivation treatment manipulation (intermittent therapy)

Prostate Cancer: Incontinence

- Pelvic muscle training with or without biofeedback
- Electrical stimulation
- Extracorporeal magnetic innervation
- Compression devices (penile clamps)
- Lifestyle changes
- Slings and artificial urinary sphincters

Prostate Cancer: Sexual dysfunction

- Couple counseling
- 5DE inhibitors daily or on-demand
- Intracavernosal injections
- Intermittent ADT instead of continuous ADT
- Penile prosthesis or pumps
