# Supplementary material for: Artificial intelligence supporting cancer patients across Europe—The ASCAPE project
Source: PLoS One. 2022 Apr 21;17(4):e0265127. doi: 10.1371/journal.pone.0265127 (PMC9022843; doi:10.1371/journal.pone.0265127)

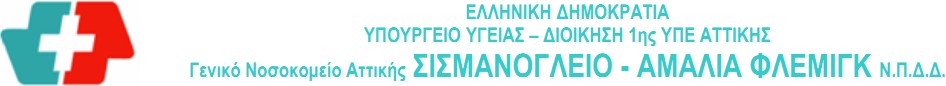


**From: Administrator’s office Date: 09/01/2020**

**To:** Lazaros Tzelves, MD (Second University Urology Department) **Protocol no: 17002**

**Subject: Approval of ASCAPE study in Second University Urology Department**

**The manager of General Hospital of Attica “Sismanoglio-Amalia Fleming”**

**approves**

the conduct of a study under the title “Prospective study to evaluate and improve the quality of life of patients with prostate or breast cancer, using artificial intelligence technology: ASCAPE study”, by Lazaros Tzelves, MD. It is a european, multi-centered, non interventional, prospective trial, aiming to accumulate wide-range data, regarding the heath-related quality of life of patients suffering from prostate or breast cancer, after they have received therapy, and the use of it, in order to train artificial intelligence models in suggesting suitable interventions to improve health-related quality of life.

One of the centers of this trial is the second Urology Department of the National and Kapodistrian University of Athens, based in Sismanoglio hospital, with Professor Ioannis Varkarakis as principal investigator.

The population of this trial will be patients with prostate cancer who receive therapy with radical prostatectomy and/or radiotherapy, and are under active surveillance afterwards, in order to accumulate data, regarding health-related quality of life.

The Sismanoglio hospital will have no financial commitment to this study, and any personal data will be insured by the existing legislation.

After the completion of this study, the investigator is obligated to file a copy to the scientific committee of the hospital.

**The Manager**

**“Sismanoglio-Amalia Fleming General Hospital of Attica”**

**Dr Ilias Dalainas**


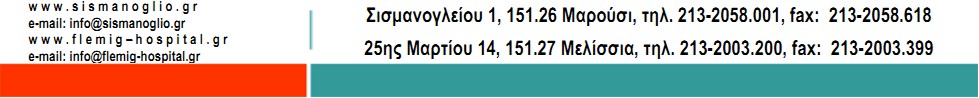

Supplement: S3 File — (DOCX) [file pone.0265127.s005.docx]
