# Supplementary material for: Artificial intelligence supporting cancer patients across Europe—The ASCAPE project
Source: PLoS One. 2022 Apr 21;17(4):e0265127. doi: 10.1371/journal.pone.0265127 (PMC9022843; doi:10.1371/journal.pone.0265127)
Supplement: S4 File — (PDF) [file pone.0265127.s006.pdf]

Ministry of Digital Governance, Hellenic Republic
Digitally signed by Ministry of Digital Governance, Hellenic Republic
Date: 2020.09.08 15:41:40 EEST
Reason:
Location: Athens

ΕΛΛΗΝΙΚΗ ΔΗΜΟΚΡΑΤΙΑ
ΥΠΟΥΡΓΕΙΟ ΥΓΕΙΑΣ – ΔΙΟΙΚΗΣΗ 1ης ΥΠΕ ΑΤΤΙΚΗΣ
Γενικό Νοσοκομείο Αττικής ΣΙΣΜΑΝΟΓΛΕΙΟ - ΑΜΑΛΙΑ ΦΛΕΜΙΓΚ Ν.Π.Δ.Δ.

ΟΡΓΑΝΙΚΗ ΜΟΝΑΔΑ «ΣΙΣΜΑΝΟΓΛΕΙΟ»

Από: Γρ. Διοικητή
-Αυτοτελές Τμήμα Ελέγχου Ποιότητας,
Έρευνας και Συνεχιζόμενης Εκπαίδευσης
- Γραμματεία Δ.Σ. Γ.Ν.Α. «Σισμανόγλειο – Αμ. Φλέμιγκ»
και του διασυνδεόμενου σε αυτό Γ.Ν. Παίδων Πεντέλης

Ημερομηνία: 1/09/2020

Αρ. Πρωτ.:17002

ΑΝΑΡΤΗΤΕΑ ΣΤΟ ΔΙΑΔΙΚΤΥΟ

ΠΡΟΣ: Ιατρό κ. Λάζαρο Τζελβέ (Β’ Πανεπιστημιακή Ουρολογική Κλινική)

ΘΕΜΑ: Έγκριση χορήγησης άδειας για τη διενέργεια μελέτης ASCAPE στη Β’ Πανεπιστημιακή Ουρολογική Κλινική

ΑΠΟΦΑΣΗ ΔΙΟΙΚΗΤΗ

Ο Διοικητής του ΓΝΑ «Σισμανόγλειο-Αμαλία Φλέμιγκ» και του διασυνδεόμενου σε αυτό ΓΝ Παίδων Πεντέλης, έχοντας υπόψη:

α) η με αρ. πρωτ. 10599/26.05.2020 αίτηση του Ιατρού κ. Λάζαρου Τζελβέ στη Νοσοκομειακή Μονάδα «Σισμανόγλειο»

β) η με αρ. πρωτ. 12700/25.06.2020 γνωμοδότηση Επιστημονικού Συμβουλίου της Νοσοκομειακής Μονάδας «Σισμανόγλειο»

γ) η με αρ. πρωτ. 13309/02.07.2020 υπεύθυνη δήλωση του Ιατρού κ. Λάζαρου Τζελβέ στη Νοσοκομειακή Μονάδα «Σισμανόγλειο»

δ) τη με αρ. πρωτ. 13821/10.07.2020 εισήγηση του Αυτοτελούς Τμήματος Ποιότητας, Έρευνας και Συνεχιζόμενης Εκπαίδευσης

αποφασίζει

την έγκριση διενέργειας μελέτης με τίτλο «Προοπτική μελέτη για την αξιολόγηση και βελτίωση της ποιότητας ζωής των ασθενών με καρκίνο του προστάτη και του μαστού χρησιμοποιώντας τεχνολογίες τεχνητής νοημοσύνης: ASCAPE study» από τον Ιατρό κ. Λάζαρο Τζελβέ. Πρόκειται για μια Πολυκεντρική, Πανευρωπαϊκή, Μη επεμβατική, προοπτική μελέτη που σκοπό έχει την συγκέντρωση δεδομένων ευρείας κλίμακας για την ποιότητα ζωής των ασθενών με καρκίνο του προστάτη και μαστού μετά τη λήψη θεραπείας και την χρήση αυτών για την εκπαίδευση μοντέλων τεχνητής νοημοσύνης ώστε να προτείνονται κατάλληλες λύσεις για την βελτίωση της ποιότητας ζωής. Το ένα κέντρο μελέτης είναι η Β’ Πανεπιστημιακή Ουρολογική κλινική του Σισμανογλείου

ΟΡΓΑΝΙΚΗ ΜΟΝΑΔΑ «ΣΙΣΜΑΝΟΓΛΕΙΟ»

Νοσοκομείου με υπεύθυνο τον Αν. Καθηγητή κ. Ιωάννη Βαρκαράκη. Ο πληθυσμός της μελέτης θα είναι ασθενείς με καρκίνο προστάτη που λαμβάνουν θεραπεία με ριζική προστατεκτομή ή/και ακτινοθεραπεία και στην συνέχεια υποβάλλονται σε ενεργό παρακολούθηση για τη συγκέντρωση δεδομένων για την ποιότητα ζωής.

Από τη διενέργεια αυτής της μελέτης δεν θα προκύψει ουδεμία οικονομική επιβάρυνση για τη νοσοκομειακή μονάδα «Σισμανόγλειο» και τυχόν προσωπικά δεδομένα θα διασφαλιστούν σύμφωνα με την κείμενη νομοθεσία.

Με το σχετικό (β) το Επιστημονικό Συμβούλιο γνωμοδότησε θετικά.

Μετά το πέρας της μελέτης, ο ερευνητής θα καταθέσει αντίγραφο στο Επιστημονικό Συμβούλιο του Νοσοκομείου.

Η παρούσα απόφαση να εισαχθεί προς επικύρωση στην επόμενη συνεδρίαση του Διοικητικού Συμβουλίου.

Ο ΔΙΟΙΚΗΤΗΣ
ΓΝΑ «Σισμανόγλειο-Αμαλία Φλέμιγκ» και
Του διασυνδεόμενου σε αυτό ΓΝ Παίδων
Πεντέλης

Δρ Ηλίας Δαλαΐνας

Εσωτερική Διανομή

1. **Διοικητικό Συμβούλιο**
2. Β' Πανεπιστημιακή Ουρολογική Κλινική
3. Αυτοτελές Τμήμα Ελέγχου Ποιότητας, Έρευνας και Συνεχιζόμενης Εκπαίδευσης
