# Supplementary material for: Artificial intelligence supporting cancer patients across Europe—The ASCAPE project
Source: PLoS One. 2022 Apr 21;17(4):e0265127. doi: 10.1371/journal.pone.0265127 (PMC9022843; doi:10.1371/journal.pone.0265127)
Supplement: S5 File — (DOCX) [file pone.0265127.s007.docx]

OPINION OF THE DRUG RESEARCH ETHICS COMMITTEE

ANA LUCIA ARELLANO ANDRINO, Secretary of the **Ethics Committee for Drug Research of the Hospital Clínic de Barcelona**

Certifies:

That this Committee has evaluated the promoter's proposal for the study to be carried out:

CODE:

DOCUMENTS WITH VERSIONS:

| Type | Sub-Type | Version |
| --- | --- | --- |
| Protocol |  | V.2 16-10-2020 |
| Patient Consent |  | V.2 16-10-2020 |

TITLE: ASCAPE-Artificial intelligence Supporting CAncer Patients across Europe

PROMOTER:

PRINCIPAL INVESTIGATOR: INMACULADA GRAU CORRAL; MONTSERRAT MUÑOZ MATEU

and considers that, taking into account the response to the clarifications requested (if any), and that:

- The necessary requirements for the suitability of the protocol in relation to the objectives of the study are met and the foreseeable risks and discomforts are justified.
- The capacity of the researcher and the available means are appropriate to carry out the study.
- That the planned economic compensation (when there is any) and its possible interference with respect for ethical principles have been evaluated and are considered adequate.
- That said study conforms to the essential ethical standards and deontological criteria that govern this center.
- That said study complies with the obligations established by the applicable investigation and confidentiality regulations.
- That said study is included in one of the lines of biomedical research accredited in this center, fulfilling the necessary requirements, and that it is viable in all terms.
- This CEI accepts that said study be carried out, and any change in the protocol or serious adverse event must be communicated to said Ethics Committee.

and states that:

**1st** At the meeting held on 10/29/2020, minutes 19/2020, it was decided to issue the report corresponding to the reference study.

**2º** The CEIm of the Hospital Clínic i Provincial, both in its composition and in its SOPs, complies with the standards of EMA / CHMP / ICH / 135/1995

**3rd** List of members:

**President**:

- JOAQUIM FORÉS I VIÑETA (Traumatologist, HCB)

**Vice president:**

- JOSEP MARÍA MIRÓ MEDA (Infectious Diseases Physician, HCB)

**Secretary:**

- ANA LUCIA ARELLANO ANDRINO (Clinical Pharmacologist, HCB)

**Members of the Board:**

- MONTSERRAT GONZALEZ CREUS (Social Worker, Customer Service, HCB)
- JOSE RIOS GUILLERMO (Statistician. Medical Statistics Platform. IDIBAPS)
- OCTAVI SANCHEZ LOPEZ (Patient Representative)
- MARIA JESÚS BERTRAN LUENGO (Epidemiologist, HCB)
- JOAQUÍN SÁEZ PEÑATARO (Clinical Pharmacologist, HCB)
- SERGI AMARO DELGADO (Neurologist, HCB) -EDUARD GUASCH CASANY (Cardiologist, HCB)
- VIRGINIA HERNANDEZ GEA (Hepatologist, HCB)
- MARINA ROVIRA ILLAMOLA (Primary Care Pharmacist, CAP Eixample)
- PAU ALCUBILLA PRATS (Clinical Pharmacologist, HCB)
- JOSE TOMAS ORTIZ PEREZ (Cardiologist, HCB) -ELENA CALVO CIDONCHA (Hospital Pharmacist, HCB) -CECILIA CUZCO CABELLOS (Nurse, HCB)
- PAULA MARTÍN FARGAS (Lawyer, HCB)
- GERMAN RODRÍGUEZ FERRER (Business Administration and Management. Planning and Strategy Management, HCB)
- SALVATORE BRUGALETTA (Cardiologist, HCB. Member of CEA, HCB)
- XAVIER CANALS-RIERA (Telecommunications Engineer)
- FRANCESC XAVIER CORBELLE (IT, HCB)
- JOSEP DÍAZ CORT (Graduate in Physical Sciences. Professor in Computer Science)
- GASPAR MESTRES ALOMAR (Medical, Angiology, Vascular Surgery, HCB)
- FRANCESC TORRALBA ROSELLÓ (Doctor of Philosophy)
- MARTA FRANCH SAGUER (Attorney)
- PATRICIA AMOROS REBOREDO (Hospital Pharmaceutical, HCB)

In the event that a project of which a member is a researcher / collaborator is evaluated, the member will be absent from the meeting during the discussion of the project. For the record, where appropriate, and at the request of the promoter,

Barcelona, ​​November 3, 2020

Reg. HCB / 2020 / 0971

Mod_04 (V4 of 06/18/2018)

HOSPITAL CLÍNIC DE BARCELONA Villaroel, 170 –

08036 Barcelona (Spain)

Tel. 93 227 54 00 Fax 93 227 54 54

[www.hospitalclinic.org](http://www.hospitalclinic.org)
