# Supplementary material for: Artificial intelligence supporting cancer patients across Europe—The ASCAPE project
Source: PLoS One. 2022 Apr 21;17(4):e0265127. doi: 10.1371/journal.pone.0265127 (PMC9022843; doi:10.1371/journal.pone.0265127)
Supplement: S6 File — (DOCX) [file pone.0265127.s008.docx]

Dnr 2021-01358

Stockholm Section 4, Medicine

**DECISION**

2021-03-31

**Applicant research principal**

Örebro Region County

**Principal investigator**

Antonis Valachis

**Title of the research project**

Implementation of Artificial Intelligence to improve quality of life in patients with cancer – ASCAPE

The Swedish Ethical Review Authority decides as below.

**DECISION**

The research project is approved with the condition that the written informed consent will be supplemented with information on the time required to complete the different parts of the project and a direct telephone number to the principal investigator.

This decision can be appealed to the Board of Appeal for ethical review. How to appeal is shown in the attached instructions.

On behalf of the Swedish Ethical Review Authority,

Peter Strömberg,

Chairman

The decision was made by the following persons:

Peter Strömberg (chairman)

**Members with scientific competence**

Göran Elinder (pediatrics, biobanks, haematology, ethical issues)

Malin Holzmann (obstetrics, fetal monitoring)

Jan Jakobsson (anesthesia, intensive care unit, acute medicine, pain)

Claudia Lampic (clinical research, psychology)

Miriam Mints (obstetrics and gynecology, obstetrik och gynekologi, rapporteur)

Erik Näslund (surgery, scientific secreterary)

**Members representing public interest**

Lena Josefsson

Dag Klackenberg

Ewa Samuelsson

Mats Wilhelmsson

**The decision is sent to**

Principal investigator: Antonios Valachis

Representative of the research principal: Mats G Karlsson
